# Functional network disruption in cognitively unimpaired autosomal dominant Alzheimer’s disease: a magnetoencephalography study

**DOI:** 10.1093/braincomms/fcae423

**Published:** 2024-11-25

**Authors:** Anne M van Nifterick, Willem de Haan, Cornelis J Stam, Arjan Hillebrand, Philip Scheltens, Ronald E van Kesteren, Alida A Gouw

**Affiliations:** Alzheimer Center Amsterdam, Neurology, Vrije Universiteit Amsterdam, Amsterdam UMC Location VUmc, 1081 HZ Amsterdam, The Netherlands; Clinical Neurophysiology and MEG Center, Neurology, Amsterdam UMC Location VUmc, 1081 HV Amsterdam, The Netherlands; Amsterdam Neuroscience, Neurodegeneration, 1081 HV Amsterdam, The Netherlands; Alzheimer Center Amsterdam, Neurology, Vrije Universiteit Amsterdam, Amsterdam UMC Location VUmc, 1081 HZ Amsterdam, The Netherlands; Amsterdam Neuroscience, Neurodegeneration, 1081 HV Amsterdam, The Netherlands; Clinical Neurophysiology and MEG Center, Neurology, Amsterdam UMC Location VUmc, 1081 HV Amsterdam, The Netherlands; Amsterdam Neuroscience, Neurodegeneration, 1081 HV Amsterdam, The Netherlands; Clinical Neurophysiology and MEG Center, Neurology, Amsterdam UMC Location VUmc, 1081 HV Amsterdam, The Netherlands; Amsterdam Neuroscience, Brain Imaging, 1081 HV Amsterdam, The Netherlands; Amsterdam Neuroscience, Systems and Network Neurosciences, 1081 HV Amsterdam, The Netherlands; Alzheimer Center Amsterdam, Neurology, Vrije Universiteit Amsterdam, Amsterdam UMC Location VUmc, 1081 HZ Amsterdam, The Netherlands; Amsterdam Neuroscience, Neurodegeneration, 1081 HV Amsterdam, The Netherlands; Amsterdam Neuroscience, Neurodegeneration, 1081 HV Amsterdam, The Netherlands; Department of Molecular and Cellular Neurobiology, Center for Neurogenomics and Cognitive Research, Vrije Universiteit Amsterdam, 1081 HV Amsterdam, The Netherlands; Alzheimer Center Amsterdam, Neurology, Vrije Universiteit Amsterdam, Amsterdam UMC Location VUmc, 1081 HZ Amsterdam, The Netherlands; Clinical Neurophysiology and MEG Center, Neurology, Amsterdam UMC Location VUmc, 1081 HV Amsterdam, The Netherlands; Amsterdam Neuroscience, Neurodegeneration, 1081 HV Amsterdam, The Netherlands; Amsterdam Neuroscience, Systems and Network Neurosciences, 1081 HV Amsterdam, The Netherlands

**Keywords:** autosomal dominant Alzheimer’s disease, magnetoencephalography, functional connectivity, hub vulnerability, neuronal network hyperexcitability

## Abstract

Understanding the nature and onset of neurophysiological changes, and the selective vulnerability of central hub regions in the functional network, may aid in managing the growing impact of Alzheimer’s disease on society. However, the precise neurophysiological alterations occurring in the pre-clinical stage of human Alzheimer’s disease remain controversial. This study aims to provide increased insights on quantitative neurophysiological alterations during a true early stage of Alzheimer’s disease. Using high spatial resolution source-reconstructed magnetoencephalography, we investigated regional and whole-brain neurophysiological changes in a unique cohort of 11 cognitively unimpaired individuals with pathogenic mutations in the presenilin-1 or amyloid precursor protein gene and a 1:3 matched control group (*n* = 33) with a median age of 49 years. We examined several quantitative magnetoencephalography measures that have been shown robust in detecting differences in sporadic Alzheimer’s disease patients and are sensitive to excitation-inhibition imbalance. This includes spectral power and functional connectivity in different frequency bands. We also investigated hub vulnerability using the hub disruption index. To understand how magnetoencephalography measures change as the disease progresses through its pre-clinical stage, correlations between magnetoencephalography outcomes and various clinical variables like age were analysed. A comparison of spectral power between mutation carriers and controls revealed oscillatory slowing, characterized by widespread higher theta (4–8 Hz) power, a lower posterior peak frequency and lower occipital alpha 2 (10–13 Hz) power. Functional connectivity analyses presented a lower whole-brain (amplitude-based) functional connectivity in the alpha (8–13 Hz) and beta (13–30 Hz) bands, predominantly located in parieto-temporal hub regions. Furthermore, we found a significant hub disruption index for (phase-based) functional connectivity in the theta band, attributed to both higher functional connectivity in ‘non-hub’ regions alongside a hub disruption. Neurophysiological changes did not correlate with indicators of pre-clinical disease progression in mutation carriers after multiple comparisons correction. Our findings provide evidence that oscillatory slowing and functional connectivity differences occur before cognitive impairment in individuals with autosomal dominant mutations leading to early onset Alzheimer’s disease. The nature and direction of these alterations are comparable to those observed in the clinical stages of Alzheimer’s disease, suggest an early excitation-inhibition imbalance, and fit with the activity-dependent functional degeneration hypothesis. These insights may prove useful for early diagnosis and intervention in the future.

## Teaser

In a resting-state magnetoencephalography study, van Nifterick *et al*. report oscillatory slowing, functional connectivity and selective hub disruption in cognitively unimpaired mutation carriers with autosomal dominant Alzheimer’s disease. The changes resemble those previously observed in clinical and sporadic Alzheimer’s disease, suggesting similar brain dysfunction mechanisms across the Alzheimer’s disease continuum.

## Graphical Abstract

**Graphical Abstract:**
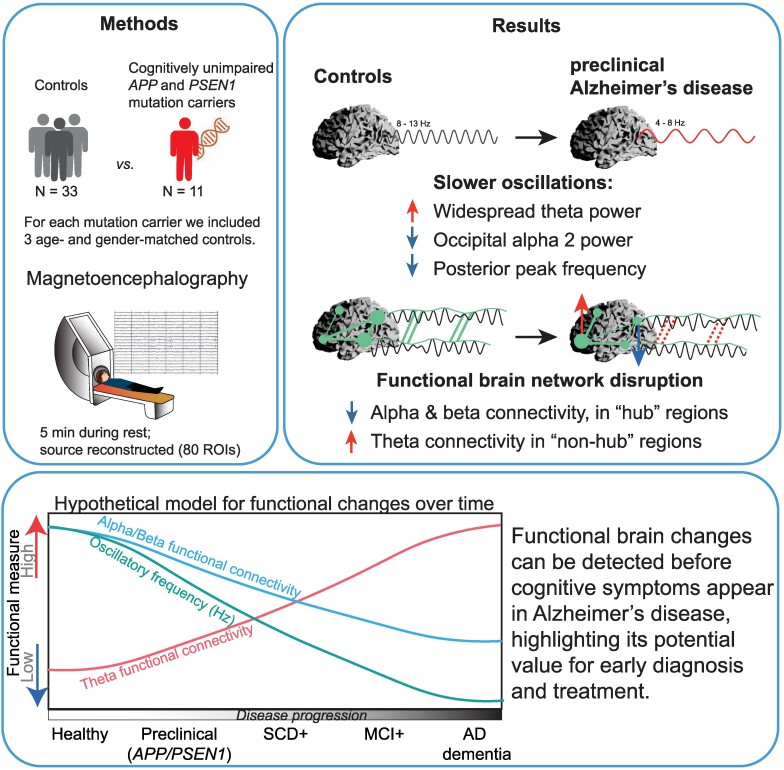

## Introduction

Alzheimer’s disease is characterized by a long pre-clinical stage with amyloid beta accumulation preceding tau pathology, neurodegeneration and cognitive impairment.^1-3^ Emerging evidence supports the hypothesis of increased excitation-inhibition ratio, particularly related to amyloid pathology, as an early and key feature in Alzheimer’s disease.^4-7^ Persistent hyperactivity of neurons is metabolically demanding and believed to contribute to functional network impairment, exacerbation of Alzheimer’s disease pathology and neurodegeneration through oxidative stress and excitotoxicity.^8-11^ This process is also referred to as ‘activity dependent degeneration’ and could explain the preferential disruption of highly connected and active hub regions in Alzheimer’s as well as other neurological diseases.^12-17^ Timely detection of abnormally increased neuronal network excitability and activity offers early diagnostic or prognostic tools, as well as a potential alternative target for non-invasive treatment at relatively low cost.^18-21^

Neurophysiological techniques provide a direct and non-invasive means to study neuronal population activity with high temporal resolution compared to other methods like functional magnetic resonance imaging (fMRI).^22^ While we currently lack robust, sensitive measures to accurately examine disrupted excitation-inhibition balance in a non-invasive manner in humans,^23-25^ research has revealed that changes in excitation-inhibition balance and network hyperexcitability manifest in quantitative neurophysiological measures, including spectral power and functional connectivity.^25-33^ Oscillatory slowing, increased functional connectivity in the lower frequency bands, and decreased functional connectivity in the higher frequency bands have been robustly observed in clinical Alzheimer’s disease patients.^34,35^ These findings suggest an excitation-inhibition imbalance, possibly due to increased excitatory neurotransmission.^6,27,31,33,36^ However, the precise macroscale neurophysiological alterations occurring during the pre-clinical stage of Alzheimer’s disease have been less frequently studied and remain controversial, due to difficulties with diagnosing AD in absence of clinical symptoms, and variations in methodologies and biomarker evidence.^35,37-46^

Alzheimer’s disease mutation carriers offer a unique opportunity to understand the timing and nature of pathophysiological changes preceding cognitive impairment.^47^ Prior electroencephalography studies in symptomatic mutation carriers have repeatedly shown oscillatory slowing.^37,45,48^ However, contradictory results have been reported for mutation carriers in the pre-symptomatic stage.^37,45,48^ These contradictions may be explained by differences in estimated years before symptom onset (EYBSO), but warrant further investigation. Furthermore, regional spectral power changes in mutation carriers has not been investigated before using high spatial resolution magnetoencephalography (MEG), allowing to detect potential subtle neuronal activity changes from deeper brain areas like the hippocampus.^28,49-51^ One EEG study reported functional connectivity increases, primarily in the precuneus, which is a hub region of the default mode network.^52^ Early hub hyperconnectivity may, thus, precede hub disruption, possibly pointing towards opposite changes in the excitation-inhibition ratio between early and late stages of Alzheimer’s disease. However, these findings were based on information-theoretic measures and limited to regions presenting differences during task-based fMRI.^52^ Functional connectivity using robust neurophysiological measures in different frequency bands and the hub disruption index (HDI) have not been investigated before in cognitively unimpaired mutation carriers.^53^

This study aims to address the need for enhancing our understanding of functional neuronal network changes in pre-clinical Alzheimer’s disease by employing MEG in cognitively unimpaired mutation carriers and matched controls. By using robust neurophysiological measures, we allow comparison to prior observations in sporadic and clinical Alzheimer’s disease patients and infer changes over the Alzheimer’s disease continuum. Furthermore, because these measures have been previously associated with neuronal network excitability and excitation-inhibition balance,^27,31,33^ our findings potentially provide insights into excitation-inhibition imbalance in pre-clinical Alzheimer’s disease, ultimately paving the way for developing early diagnostic or prognostic tools and targeted treatments.

## Materials and methods

### Study population

Cross-sectional observational data utilized in the present study was collected at the Amsterdam UMC, location VUmc, through a combination of MEG recordings, MRI scanning and clinical assessments. Part of this dataset has been previously analysed.^54^ The dataset comprised 11 subjects with autosomal dominant mutations in either the *PSEN1* (*n* = 9) or *APP* (*n* = 2) genes. Genetic testing was part of clinical care and performed after counselling by a clinical geneticist. The majority of genetic tests were carried out at Amsterdam UMC, location VUmc. If in a family the specific mutation was known from other affected family members, only the specific mutation was tested using Sanger sequencing. *APP* duplication and *PSEN1* deletions were measured with commercial multiplex ligation-dependent probe amplification (MLPA) (P170-C3 and P254-B2, MRC Holland). If there was no known mutation in the family, individuals were tested with an exome-based gene panel supplemented with MLPA of *PSEN1* and *APP* as well as *C9ORF72* repeat length measure. More details about the whole-exome sequencing are included in the Supplementary materials. We included *APP* and *PSEN1* mutation carriers who exhibited no cognitive impairment, as confirmed by extensive neuropsychological examination (Supplementary material). For each mutation carrier, three sex- and age-matched healthy controls (total *n* = 33) with available five-minute eyes closed resting-state MEG and structural MRI were included from other study cohorts of the Amsterdam UMC (specifically, MANTA (2018.070), EMIF-AD (2014.210), the MuMo Brain project (2018.330), the Amsterdam Dementia Cohort (2016.061) and the Amsterdam MS Cohort).

Whenever possible, we matched controls to mutation carriers on additional factors including MEG scanner type, educational level and Mini-Mental State Examination (MMSE) scores. Control subjects were selected based on the absence of cognitive, neurological or psychiatric disorders, as well as the non-use of psychoactive medication at time of measurement, and negative amyloid-beta status if available (determined by amyloid positron-emission tomography or cerebrospinal fluid examination).

The study protocol was approved by the medical ethical committee of the Amsterdam UMC, location VUmc and was conducted in accordance with the principles in the declaration of Helsinki. All participants provided written informed consent to inclusion in the study.

### Estimated years before symptom onset

The EYBSO was calculated as the difference (in years) between the mutation carrier’s age and (i) the reported parental age at symptom onset (or their affected sibling age at onset if the former was not known) (EYBSO_parent), (ii) the mean age at onset for all family members (EYBSO_family) and (iii) the mean age at onset for all individuals with a similar mutation type as reported in the literature (EYBSO_mutation).^47^

### Clinical assessments

To ascertain that mutation carriers were cognitively unimpaired, MMSE and an extensive neuropsychological test battery were performed (Supplementary material). Furthermore, several self-reported or informant-based cognitive complaints were assessed using the subjective cognitive functioning (SCF) questionnaire,^55^ the cognitive change index (CCI),^56^ and a short version of the Amsterdam questionnaire measuring ‘instrumental activities of daily living’ in (early) dementia^57^ (Supplementary material). The Hospital Anxiety and Depression Scale^58^ was also obtained to take into account for potential interference of a major anxiety or depressive disorder with cognition.

### MEG recordings

MEG recordings were performed in a magnetically shielded room on two different MEG scanners. For 8 mutation carriers and 29 controls, 5 min of eyes closed resting-state MEG was recorded using a 306-channel whole-head Vectorview system (Elekta Neuromag Oy, Helsinki, Finland) at a sample frequency of 1250 Hz, an online anti-aliasing filter of 410 Hz and high-pass filter of 0.1 Hz. In April 2021, the MEG system was replaced by a Triux Neo system (MEGIN Oy, Finland) with identical channel number and type, allowing combined use of data. MEG was acquired without internal active shielding^59^ using the Triux Neo system for three mutation carriers and four controls, at a sampling frequency of 1000 Hz, an online anti-aliasing filter of 330 Hz and high-pass filter of 0.1 Hz.

To determine the head position relative to the MEG sensors, five head-position indicator coils and the outline of the participant’s scalp and nose were digitized using a 3D digitizer (Fastrak, Polhemus, Colchester, VT, USA). All subjects were in a comfortable supine position during MEG recording and were instructed to close their eyes, lie still and relax while staying alert. An experienced technician continuously monitored the recording and alerted the patient by signs of drowsiness through an acoustic signal or instructing the patient to shortly open the eyes.

### MEG pre-processing

MEG data were processed to obtain artefact-free source-level data of 80 regions (78 cortical regions and 2 hippocampi) of the automatic anatomical labelling (AAL) atlas (Supplementary Table S1).^60^ Channels with excessive artefacts (e.g. flat lines and squid-jumps) were excluded before estimation of the temporal extension of signal space separation coefficients^61^ implemented in MaxFilter software (Elekta Neuromag Oy, version 2.2.15) to suppress environmental noise. MEG data were co-registered to the individual T1-weighted structural MRI scan. MEG recording was performed before or at least 1 week after MRI scanning to avoid potential magnetization artefacts. The digitized scalp outline was co-registered with the subject’s structural MRI using surface matching (based on the iterated closest point technique in combination with an in-house developed software). The sphere that best fitted the individual scalp surface was used as a volume conductor model. Following broad band filtering (0.5–100 Hz) and excluding the first second and last 10 s of the time-series, an atlas-based centroid beamforming approach was applied as previously described^49,50,62^ to obtain source-level MEG time-series. The equivalent current dipole as source model, spherical volume conductor model and the MEG data covariance matrix were used to compute the broad band beamformer weights, using a scalar beamformer as implemented in Elekta beamformer software (version 2.1.28). Each voxel of the co-registered MRI was labelled to one of the 80 regions and the beamformer-reconstructed time-series of the centroid voxel of each region was used as the representative source. These source-reconstructed time-series were converted to ASCII files for visualization and further analyses in an in-house developed Java-based package, BrainWave (version 0.9.163.22, https://github.com/CornelisStam/BrainWave).

### Data selection

The source-reconstructed MEG data were downsampled to 312 Hz and segmented in epochs of 13.11 s (Vectorview data), or to 333 Hz in epochs of 12.29 s (Triux Neo data), containing 4096 samples. For each subject, 10 non-overlapping, eyes-closed epochs of the best quality were selected, based on careful visual inspection by AN and/or an experienced neurologist, not directly involved in the analysis. All subsequent analyses were performed for each region, each frequency band and each epoch separately. Results were averaged across epochs to obtain one value per region per subject. Whole-brain average results were obtained through averaging across all regions. For controls, all MEG outcome measures were averaged over three subjects who matched to one mutation carrier to obtain a robust ‘surrogate control’ value.

### Spectral power analyses

A broad band power spectrum (0.5–48 Hz, with a frequency resolution of 0.076 Hz for Vectorview data and 0.081 for Triux Neo data), was obtained through Fast Fourier transformation of the time-series, from which absolute and relative spectral power were computed for six frequency bands (delta (0.5–4 Hz), theta (4–8 Hz), alpha 1 (8–10 Hz), alpha 2 (10–13 Hz), beta (13–30 Hz) and gamma (30–48 Hz)). The peak frequency was defined as the frequency at which power was maximum within the extended alpha band (4–13 Hz).

### Functional connectivity analyses

Functional connectivity for each combination of two source-reconstructed time-series was estimated in the theta band, using the phase lag index (PLI),^63^ and in the alpha (8–13 Hz) and beta band, using the amplitude envelope correlation metric corrected for volume conduction (AECc).^64,65^ Previous work has shown the reliability of these measures in healthy subjects,^66^ and robustness in these specific frequency bands to capture differences between AD patients and controls in source-space MEG data of comparable epoch length.^53^

In short, the instantaneous phase and amplitude (envelopes) were estimated using the Hilbert transform. The PLI calculates the asymmetry of the distribution of instantaneous phase differences between two time-series. The PLI is insensitive to shared signals at zero phase lag and, thus, by construction, excludes connectivity between regions due to signal leakage/volume conduction. PLI values range between 0 and 1, where 0 indicates no (or zero-lag) coupling and 1 refers to perfect (non-zero-lag) coupling.

The AEC is obtained by a Pearson correlation of the amplitude envelopes of two time-series. A pair-wise orthogonalization in two directions, by means of a linear regression, is performed to correct for signal leakage, meaning that time-series X is regressed out from time-series Y and the other way around, before correlation analyses. The correlation values for both directions are subsequently averaged per pair of time-series. The AECc values were normalized by adding one and then divide by two to obtain AECc values in the range [0–1], where 0.5 indicates no coupling.

The symmetric 80 × 80 connectivity matrices were averaged over epochs and across rows to obtain a single functional connectivity strength value per region per subject. This is also known as the weighted functional degree and represents a measure of centrality, or hubness, of a region in the network. In addition, the functional connectivity values were averaged across all pairs of regions (i.e. across all rows and columns) to obtain a single, whole-brain average, functional connectivity value per subject.

### Hub vulnerability analyses

To identify whether regional differences in functional connectivity depend on the role of a region in the brain network (i.e. central hubs versus peripheral regions) and associated level of neuronal activity,^13,15,17^ we computed the HDI.^67,68^

First, HDI analyses include subtraction of the weighted functional degree of a region in the reference (control) network from the degree of the respective region in a target network (i.e. mutation carriers). Second, we plot this regional difference as a function of the functional degree of the respective region in the reference network. Third, we fit a linear regression line to this data.

HDI analyses was performed for each connectivity measure (*n* = 2) and frequency band (*n* = 3) and using GraphPad Prism software (version 9.5.1). First, we determined HDI analyses for mutation carriers in two ways: (i) we used group average regional functional connectivity results to compare mutation carriers to controls on a group-level, and (ii) we performed HDI analyses on an individual level, so for each pair of mutation carrier–surrogate control, to take the matching into account. Second, similar as for the mutation carriers, we also determined the individual HDI for each control compared to the mean of their surrogate control group (*n* = 3).

If the linear regression line is significantly different from zero, it means that the difference in functional degree depends on the reference degree. A negative slope (HDI < 0) indicates a specific disruption of hubs, and/or a specific increase in functional connectivity in low degree, non-hubs depending on the offset. If the opposite is true, we would find significant positive slopes (HDI > 0), and if there is no degree-dependent change, the regression line would be horizontal and non-significant (HDI ± 0).

### Statistical analyses

All statistical analyses were performed using non-parametric tests, because of the low number of subjects. Group-level demographics and clinical characteristics were compared with Mann–Whitney U-tests or Pearson chi-square (*χ*^2^) tests where appropriate, using IBM SPSS statistics (version 28, Armnk, NY: IBM Corp).

Due to the careful matching between mutation carriers and surrogate controls, we employed pairwise comparisons to analyse MEG outcome measures. This approach allows to directly compare each mutation carrier with a corresponding surrogate control, thus reducing the standard error. By minimizing variability between pairs, this method enhances statistical power, increasing the likelihood of detecting true effects.

For whole-brain average MEG data, we used a Wilcoxon matched-pairs signed rank sum test as integrated in IBM SPSS Statistics, version 28. A *P* < 0.05 was considered statistically significant.

Regional comparisons were performed using a paired-sample permutation test (through 2000 permutations) incorporated in an in-house developed MATLAB script written by AH and corrected for multiple comparisons using the maximal statistic (*P* < 0.05).^69^ We reported both uncorrected and corrected regional statistical test results because multiple comparisons correction may be too strict in this small study cohort. To prevent over-interpretation of results, uncorrected significant regional test results were evaluated in the discussion section only if the whole-brain average test results were significant.

Despite the low sample size, we performed Spearman correlation coefficient analyses to gain insights into how MEG measures change across the pre-clinical disease stage, possibly driven by rising amyloid accumulations. We selected a total of seven MEG measures, including those showing significant whole-brain results (*n* = 4) and all individual hub disruption indices (*n* = 3). We included a total of seven clinical variables in the correlation analyses as markers for the stage of progression within the pre-clinical phase of the disease: age, three measures of the EYBSO (parent, family and mutation), the MMSE, the Rey auditory verbal learning test (RAVLT) delayed recall *t*-scores (adjusted for age and sex), and the measure of self-reported cognitive complaints with the largest interquartile range, i.e. the self-reported CCI. The RAVLT delayed recall was specifically selected because it is similar to the word list recall memory test in the Consortium to Establish a Registry for Alzheimer’s Disease battery, a sensitive measure of cognitive change in pre-dementia mutation carriers.^70^

To better understand if potential correlations between MEG measures with age and EYBSO were due to disease progression rather than ageing, we performed similar correlation analyses within the control group (*n* = 33). Correlation analyses were conducted in SPSS and a *P*-value < 0.05 was considered statistically significant. We applied a false discovery rate (FDR) correction using the two-stage linear step-up procedure of Benjamini, Krieger and Yekutieli to adjust the *P*-values, with a significance level set at 0.05. The FDR correction was performed in GraphPad Prism software (version 9.5.1).

### *Post-hoc* analyses

To reduce the risk of outliers influencing the non-parametric test results, we inspected whole-brain average MEG pair-wise differences. Suspected outliers were determined as those data points that were more than 1.5 times the interquartile range away from the first or third quartile. Suspected outliers were examined with the aim to determine the source and, if needed, were removed before repeating the analyses (*post-hoc*).

## Results

### Subject characteristics

Due to careful matching, mutation carriers and controls did not differ in demographic characteristics (Table 1). A number of mutation carriers already passed their estimated age of symptom onset (*n* = 6), and one mutation carrier exhibited mild attention problems, but none showed cognitive impairment on neuropsychological examination (Supplementary Table S2). One mutation carrier self-reported an abnormal CCI, which could be associated to the high level of self-reported anxiety.

**Table 1:** Subject characteristics

	Mutation carriers (*n* = 11)	Controls (*n* = 33)
Age (years)	49 [20–61]	49 [20–62]
Female/male (*n*)	8/3	24/9
*PSEN1/APP* (*n*)	9/2	–
Education (Verhage)	6 [5–7]	6 [1–7]
Global cognition (MMSE)	29 [27–30] (*n* = 11)	27 [27–30] (*n* = 7)
EYBSO (y)		
Parental	1 [−16–22]	–
Family	4 [−8–32]	–
Mutation	3 [−12 27]	–
MEG system Elekta/MEGIN (*n*)	8/3	29/4

Group median and range [min–max] are presented, unless otherwise specified. *PSEN1*, presenilin-1 gene mutation; *APP*, duplication of the amyloid precursor protein (*APP*) gene; Education is presented in Verhage score (range 1–7); MMSE, Mini-Mental State Examination (max 30); EYBSO, estimated years before symptom onset; a negative EYBSO indicates that the subject passed the estimated age of symptom onset.

### Spectral slowing in cognitively unimpaired *APP* and *PSEN1* mutation carriers

#### Whole-brain

**Figure 1:**
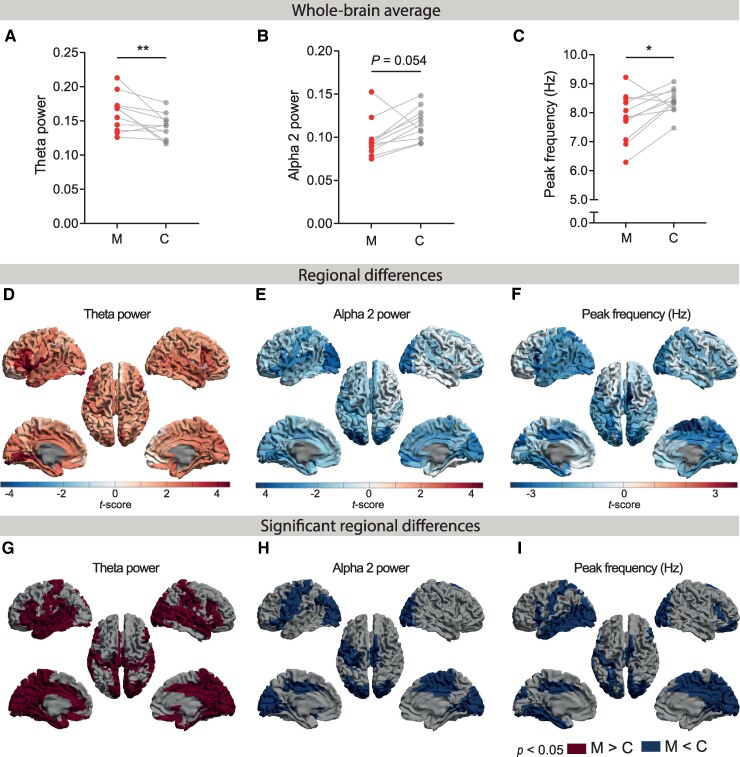
**Spectral activity in Alzheimer’s disease mutation carriers and controls.** (**A-C)** Each data point displays the whole-brain average MEG-based spectral measure for mutation carriers (M, *n* = 11) in red and (surrogate) age- and gender-matched controls (**C**, *n* = 11) in grey. Relative power was calculated as the power within a specific frequency band divided by the total power across all bands (0.5–48 Hz). Wilcoxon matched-pairs rank sum tests revealed a relative power difference in the theta and alpha 2 frequency bands, and a different peak frequency between mutation carriers and (surrogate) controls. (**D-F)** A paired permutation test was conducted to compare spectral brain activity between mutation carriers (*n* = 11) and (surrogate) controls (*n* = 11) across 80 different brain regions. The brain colour plots illustrate the regional *t*-scores based on these tests. Warmer and colder colours indicate higher and lower levels in mutation carriers compared to (surrogate) controls, respectively. (**G-I)** Thresholded brain colour maps show regions with significantly different *t*-scores (*P* < 0.05) (without multiple comparisons correction) between mutation carriers and (surrogate) controls. M, mutation carrier; C, control; *, *P* < 0.05; **, *P* < 0.01.

Based on the Wilcoxon matched-pairs signed rank sum test, we found a significantly higher whole-brain average relative theta power in mutation carriers (Median (*Mdn*) [min–max] = 0.168 [0.126–0.213]) compared to controls (*Mdn* = 0.143 [0.117–0.177]), *z* = −2.490, *P* = 0.014 (Fig. 1A). In the remaining sections of this report, we will refer to and report ‘relative’ power values without explicitly using the term ‘relative’. At the group level, alpha 2 power was lower at trend level for mutation carriers (*Mdn* = 0.093 [0.075 −0.153]) than for controls (*Mdn* = 0.115 [0.093–0.148]), *z* = −1.956, *P* = 0.050 (Fig. 1B). Peak frequency was significantly lower in mutation carriers (*Mdn* = 7.85 Hz [6.29–9.22 Hz]) compared to controls (*Mdn* = 8.39 Hz [7.47–9.07 Hz]), *z* = −2.134, *P* = 0.033 (Fig. 1C). Other frequency bands did not show significant group differences (Supplementary Fig. S1, ‘left column’). These findings demonstrate oscillatory slowing in cognitively unimpaired mutation carriers at whole-brain level.

#### Regional

A paired-samples permutation test showed higher theta power in 50 regions in mutation carriers compared to controls, involving both the left and the right temporoparieto-occipital, frontal and cingulate cortex, insula and hippocampi (Fig. 1D and G; Supplementary Table S3). Alpha 2 power was significantly lower in 27 regions, involving somewhat more regions in the left compared to the right hemisphere (*n* = 17 and *n* = 10, respectively), including regions in the occipital cortex as well as orbitofrontal, central, parietal and middle cingulate cortices (Fig. 1E and H; Supplementary Table S3).

Peak frequency was significantly lower in 26 regions, involving regions in the left but not right temporal cortex, bilateral parieto-occipital cortices, the middle and posterior cingulate cortices, both hippocampi, the right supplementary motor area, and right orbitofrontal cortex (Fig. 1F and I; Supplementary Table S3).

While delta, alpha 1 and beta power did not differ between groups on whole-brain level, groups differed on a regional level (Supplementary Fig. S1 and Table S3). Delta power was higher in mutation carriers in 13 regions, involving occipito-parietal and central cortices such as the right precuneus, the left and right posterior cingulate cortices, and the right medial orbital frontal cortex. Delta power was significantly lower in the right supplementary motor area.

Alpha 1 power was significantly lower in 4 regions of the right hemisphere, involving the frontal and temporoparietal cortices such as the right orbitofrontal cortex. Alpha 1 power was significantly higher in the right supplementary motor area. Beta power was significantly lower in 21 regions, involving somewhat more regions in the right compared to the left hemisphere (*n* = 13 and *n* = 8, respectively), including bilateral insula, anterior cingulate and frontal cortical regions such as the medial orbitofrontal gyri and triangular part of the frontal inferior gyrus, and the right superior temporal pole. Gamma power did not differ between groups.

Higher theta power in the left insula, lingual gyrus and triangular part of the frontal inferior gyrus, and lower alpha 2 power in the left superior occipital cortex were also significant after multiple comparisons correction (Supplementary Fig. S1 and Table S3). Taken together, mutation carriers exhibit higher theta power in multiple occipitotemporal, insular and frontal regions and lower alpha 2 power primarily in the occipital cortex.

### Functional connectivity disruption in cognitively unimpaired *APP* and *PSEN1* mutation carriers

#### Whole-brain

**Figure 2:**
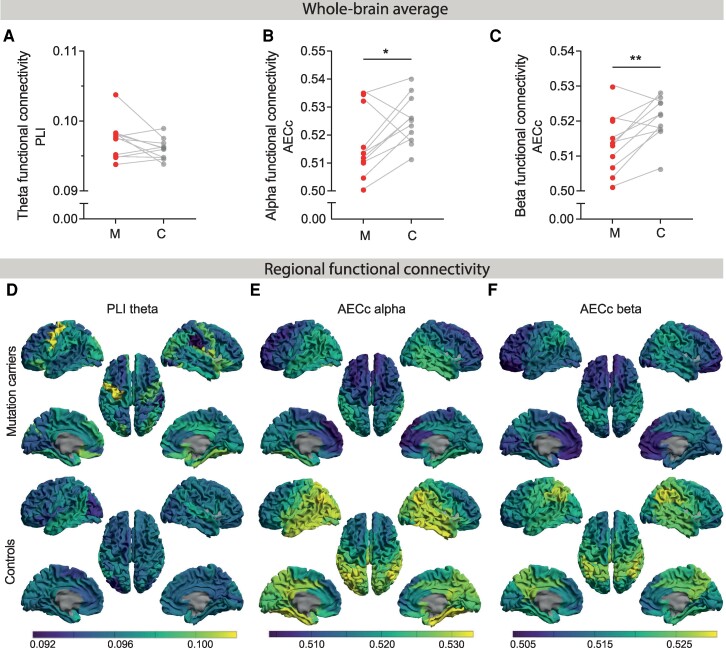
**Functional connectivity in Alzheimer’s disease mutation carriers and controls.** (**A-C)** Each data point displays the whole-brain average MEG-based functional connectivity per mutation carrier (M, *n* = 11) in red and for each (surrogate) control (C, *n* = 11) in grey. Wilcoxon matched-pairs signed rank sum tests revealed lower functional connectivity in the alpha and beta band (AECc) in mutation carriers compared to (surrogate) controls. (**D-F)** Brain colour plots illustrate the average regional functional connectivity for 78 cortical regions in 11 mutation carriers (top row) and 11 (surrogate) controls (bottom row). Lighter (yellow) and darker (blue) colours indicate relatively high and low functional connectivity levels, respectively. PLI, phase lag index; AECc, amplitude envelope correlation corrected for volume conduction; *, *P* < 0.05; **, *P* < 0.01.

To assess functional connectivity, PLI was measured in the theta band and AECc in the alpha and beta bands. Based on the Wilcoxon matched-pairs signed rank sum test, we found no significant difference in whole-brain average theta band PLI (Fig. 2A). Alpha band AECc was significantly lower in mutation carriers (*Mdn* = 0.513 [0.500–0.535]) compared to controls (*Mdn* = 0.525 [0.511–0.540]), *z* = −2.045, *P* = 0.041 (Fig. 2B). Whole-brain average beta band AECc was significantly lower in mutation carriers (*Mdn* = 0.514 [0.501–0.530]) compared to controls (*Mdn* = 0.522 [0.506–0.528]), *z* = −2.578, *P* = 0.010 (Fig. 2C). Mutation carriers, thus, have lower whole-brain average amplitude-based functional connectivity in higher frequency bands.

#### Regional

The strength of functional connectivity (weighted degree) varied across the brain in both mutation carriers and controls (Fig. 2D-F). Hub locations, i.e. regions with highest weighted degree, within controls depended on functional connectivity measure and frequency band: theta band PLI revealed hub locations in temporoparietal regions, alpha band AECc in temporoparietal, cingulate and occipital regions, and beta band AECc in temporoparietal, cingulate and postcentral regions (see also Supplementary Table S5).

A paired-samples permutation test demonstrated that the theta band PLI was higher in 11 bilateral regions, involving the frontal, occipital, cingulate and temporal cortices as well as the insula, and was lower in the right supramarginal gyrus in mutation carriers compared to controls (Supplementary Fig. S2A and D and Table S4). The alpha band AECc was lower in 36 regions, involving more regions in the right compared to the left hemisphere (*n* = 25 and *n* = 11, respectively) and primarily located in anterior regions as well as the right hippocampus, posterior cingulate and some regions of the temporoparietal cortex, but also involving the bilateral middle orbitofrontal and anterior-to-middle cingulate cortices (Supplementary Fig. S2B, C, E, and F and Table S4). The beta band AECc was lower in 66 bilateral regions, involving the temporoparietal, frontal anterior-to-posterior cingulate and central cortices.

After multiple comparisons correction, regional theta band PLI was not different between groups (Supplementary Fig. S2G and Table S4), but two bilateral mid-and inferior-frontal regions demonstrated significantly lower alpha band AECc (Supplementary Fig. S2H and Table S4), and 23 regions showed a significantly lower beta band AECc, including the bilateral frontal, central and anterior-to-posterior cortices, along with the right precuneus (Supplementary Fig. S2I and Table S4). Together, functional connectivity results present theta band PLI differences with variable directions across regions, and predominantly lower alpha and beta band AECc in anterior regions, with additional lower beta band AECc in the cingulate cortex and precuneus.

#### Hub vulnerability

**Figure 3:**
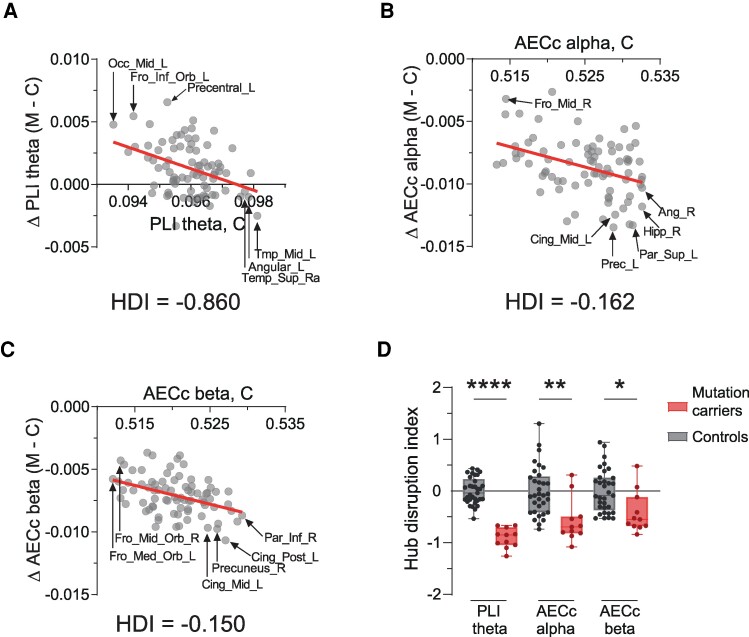
**HDI within Alzheimer’s disease mutation carriers.** (**A-C**) Each data point displays the absolute difference in regional average weighted functional degree between mutation carriers (*n* = 11) and (surrogate) controls (*n* = 11) (on the *y*-axis), presented as a function of the average functional degree of the corresponding region in (surrogate) controls (*n* = 11, on the *x*-axis), for PLI theta (**A**), AECc alpha (**B**) and AECc beta (**C**). A linear regression analyses indicated whether regional differences were significantly dependent on the functional degree (or ‘hubness’) of the corresponding region in controls. The slope of this linear regression line represents the HDI. (**D)** Bar graph with individual HDI values for mutation carriers and individual controls. A Wilcoxon matched-pairs signed rank sum test presented significant differences in HDI values between mutation carriers (*n* = 11) and controls (*n* = 33). HDI, hub disruption index; PLI, phase lag index; AECc, amplitude envelope correlation corrected for volume conduction; M, mutation carriers; C, controls; *, *P* < 0.05; **, *P* < 0.01; ***, *P* < 0.001; **** *P* < 0.0001.

To investigate whether hub regions are especially vulnerable to functional connectivity changes in mutation carriers, we computed the HDI. Group-level HDI analyses revealed a significant relationship between the average functional degree difference between mutation carriers and controls and ‘hubness’ of the corresponding region within controls (Fig. 3A-C; Supplementary Table S6). Regions with large differences tended to be located in anterior non-hub regions such as the left orbitofrontal and pre-central gyrus for theta band PLI (Fig. 3A; Table 2) and in posterior hub regions, such as the precuneus and the middle and posterior cingulum, for both alpha and beta band AECc (Fig. 3B and C; Table 2).

**Table 2:** Region with the largest absolute functional connectivity difference between mutation carriers and controls

	Largest increase (M > C)		Largest decrease (M < C)			
	PLI theta		AECc alpha		AECc beta	
Group-level						
	Central	Precentral_L	Parietal	Precuneus_L	Cingulate	Cingulum_Post_R
Subject-level						
	Frontal	Frontal_Inf_Orb_L	Parietal	Parietal_Sup_L	Parietal	Parietal_Inf_R
		Frontal_Sup_Medial_L		Parietal_Inf_R		Angular_R
		Rectus_R		SupraMarginal_R	Occipital	Precuneus_R
		Frontal_Inf_Orb_R		Precuneus_R		Occipital_Sup_R
		Frontal_Inf_Orb_R	Occipital	Calcarine_L		Occipital_Inf_R
	Central	Supp_Motor_Area_L		Occipital_Mid_R	Central	Rolandic_Oper_L
		Precentral_L		Cuneus_R		Postcentral_R
		Precentral_L	Central	Precentral_L	Frontal	Frontal_Mid_L
		Precentral_L		Postcentral_R		Frontal_Sup_Medial_L
	Temporal	Temporal_Mid_R	Temporal	Heschl_L	Temporal	Fusiform_R
		Heschl_R	Hipp	Hippocampus_R	Cingulate	Cingulum_Post_R

This table shows the region and corresponding brain lobe with the largest absolute functional connectivity difference between mutation carriers and controls on group-level and subject-level (for each mutation carrier (*n* = 11) compared to its surrogate control). For theta band PLI, we presented those regions with the largest increase and for alpha and beta band AECc we presented those with the largest decrease. M, mutation carrier; C, control.

To take the matching into account, individual HDI’ were also calculated. This revealed similar relationships and a significant HDI for theta band PLI (mean individual HDI = 0.91) in each mutation carrier (Supplementary Fig. S3 and Table S6). For alpha (mean individual HDI = −0.58) and beta band AECc (mean individual HDI = −0.42), significant ‘negative’ HDI’s (i.e. negative slopes) were found in a majority of the mutation carriers (*n* = 9 and *n* = 8 out of 11, respectively). These negative HDI’s reflected a selective hub disruption, although in one subject this was driven by an increase in functional degree in non-hub regions for alpha band AECc. One out of 11 mutation carriers showed a significant ‘positive’ HDI for alpha band AECc, driven by a decrease in functional degree in non-hub regions. One out of 11 mutation carriers showed a significant ‘positive’ HDI for beta band AECc, driven by an increase in functional degree in hub regions (Supplementary Figs. S4 and S5 and Table S6). One out of 11 mutation carriers showed no significant HDI for alpha band AECc and two out of 11 mutation carriers showed no significant HDI for beta band AECc. The individual regions with the largest difference between mutation carriers and controls were frequently located in the frontal and central cortex for theta band PLI and distributed across occipital and temporoparietal regions for alpha and beta band AECc (Table 2).

We also determined the individual HDI for each control (*n* = 33) using their surrogate control (*n* = 3) group as reference. While six controls showed a significant HDI for theta band PLI, 23 for alpha band AECc, and 24 for beta band AECc, the slopes were both positive and negative and the mean HDI’s approximately zero (Supplementary Fig. 6A-C).

A Kruskall–Wallis and *post-hoc* Dunn’s multiple comparisons test showed significant group differences between the individual HDI’s for mutation carriers and controls (*H*(6) = 47.95, *P* < 0.001; Dunn’s multiple comparisons test: theta band PLI: *z* = 5.366, *P* < 0.001; alpha band AECc: *z* = 3.415, *P* = 0.002; beta band AECc: *z* = 2.620, *P* = 0.026) (Fig. 3D). This indicates that healthy controls do (indeed) not have a significant disruption of hubs, in contrast to cognitively unimpaired mutation carriers who show a significant HDI, predominantly for theta PLI but also for AECc in higher frequency bands.

#### Association between MEG measures and cognitive indices

**Figure 4:**
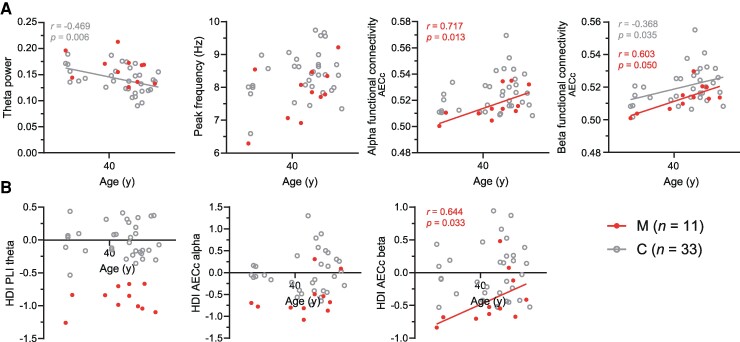
**Correlations between age and MEG measures within mutation carriers and controls.** (**A**) Each data point in the scatter plots displays a whole-brain average MEG measure for mutation carriers (M) in red (*n* = 11) and controls (C) in grey (*n* = 33), presented as a function of age. (**B**) The same as for (**A**) but then showing the HDI, measured with theta band PLI, and alpha and beta band AECc, as a function of age for mutation carriers (*n* = 11) and controls (*n* = 33). Simple linear regression lines are shown for mutation carriers and controls separately when a Spearman correlation analyses revealed significant correlations (*P* < 0.05) between MEG outcome and age. PLI, phase lag index; AECc, amplitude envelope correlation corrected for volume conduction.

Spearman’s rho correlation analyses revealed a positive correlation within mutation carriers between age and functional connectivity measured with both whole-brain average alpha band AECc (*r* = 0.717, *P* = 0.013) and beta band AECc at trend level (*r* = 0.603, *P* = 0.050), and a significant positive correlation between age and HDI beta band AECc (*r* = 0.644, *P* = 0.033). Similarly, correlation analyses revealed a negative correlation between EYBSO_parent and both whole-brain average beta band AECc (*r* = −0.670, *P* = 0.024) and HDI beta band AECc (*r* = −0.624, *P* = 0.040). A younger age and more time to symptom onset were, thus, associated with lower functional connectivity strength in the alpha and beta bands, and a stronger hub disruption in the beta band (Fig. 4A and B). Repeating these analyses using distinct calculation methods for EYBSO showed a comparable (trend for) correlations between EYBSO and beta band AECc (for whole-brain and HDI measures) (Supplementary Table S7).

Correlation analyses between age and whole-brain average MEG measures within controls revealed a negative association between age and theta power (*r* = −0.468, *P* = 0.006) and a positive association between age and beta band AECc (*r* = 0.368, *P* = 0.035) (Fig. 4A). The individual HDI did not correlate with age. As age increases, theta power showed a tendency to decrease while beta band AECc showed a tendency to increase, highlighting distinct age-related trends in neuronal oscillatory activity with advancing age within the control group.

None of the investigated cognitive test scores (MMSE and RAVL delayed recall) or self-reported change in cognition (CCI) correlated with MEG measures in mutation carriers or controls. After multiple comparisons correction, no significant associations were found in mutation carriers or controls.

### *Post-hoc* analyses

We identified one outlier in whole-brain average alpha 2 power when comparing pair-wise differences, which was attributed to an extreme value observed within a mutation carrier. Additional inspection revealed normal clinical characteristics but a cluster of microbleeds and superficial siderose in the left occipital cortex, as visualized on T2* sequence MRI of the brain (Supplementary Methods). None of the other mutation carriers presented abnormalities on (FLAIR) MRI. After excluding this carrier-control pair, we found comparable but stronger group differences (Supplementary Results). This included a stronger group-wise HDI for alpha band AECc and a higher number of regions showing significant differences, particularly in alpha 2 power, peak frequency and alpha band AECc (Supplementary Table S8).

## Discussion

By employing source-reconstructed MEG in cognitively unimpaired individuals carrying mutations in the *APP* and *PSEN1* genes, this study aimed to provide increased insights on quantitative neurophysiological alterations during a true early stage of Alzheimer’s disease. We found widespread slowing of oscillatory activity and differences in functional connectivity with a selective vulnerability of hub regions in mutation carriers compared to age-and gender-matched controls. Slowing of oscillatory activity was reflected by higher relative theta power, involving temporal and frontal regions, and lower relative alpha 2 power and peak frequency, particularly in occipital and parieto-temporal regions, respectively. Network hubs showed lower alpha and beta band amplitude-based functional connectivity as well as lower theta band phase-based connectivity, whereas non-hub regions showed higher theta band phase-based funtional connectivity. These results align well with previous observations in sporadic Alzheimer’s disease patients and highlight that abnormal neuronal function is detectable in the pre-clinical stage using non-invasive quantitative MEG measures. Because both oscillatory slowing and specific functional connectivity differences have been associated with excitation-inhibition imbalance, these results suggest such an imbalance is an early signature of Alzheimer’s disease.

### Oscillatory slowing in cognitively unimpaired mutation carriers

On the one hand, the spectral power results fit well with a robust gradual oscillatory slowing profile in Alzheimer’s disease, starting from a pre-clinical stage.^2,71-73^ Oscillatory slowing may reflect increased excitatory neuronal activity and excitability, as indicated by various studies including computational modelling studies.^6,31,32,74^ Amyloid starts to accumulate around 25 years before estimated symptom onset in regions such as the precuneus, posterior cingulate gyrus and medial orbitofrontal regions^75,76^ and has been frequently associated with increased neuronal excitability in experimental work,^4,5,77-80^ possibly contributing to the observed global and regional spectral changes from a very early disease stage.

On the other hand, a number of studies found results in the opposite direction, i.e. an acceleration in oscillatory activity reflected by higher relative alpha 2 power both in the mild cognitive impairment (MCI) stage of sporadic Alzheimer’s disease,^38,39^ and in pre-symptomatic *PSEN1* mutation carriers (at the mean age of 29 and 35 years) with additional lower theta power, predominantly in the precuneus.^37^ Furthermore, the theta/alpha2 ratio positively correlated with age in *PSEN1* mutation carriers specifically and was associated with the conversion from the asymptomatic to the symptomatic stage.^37^ The divergent findings, i.e. an initial acceleration and later (but still pre-clinical) slowing, may, therefore, be explained by age and amyloid pathology differences.^40,71^ The previously reported interneuron dynamics may provide a potential explanation for the paradoxical results among pre-clinical mutation carriers at different age, showing also dynamic changes along the early disease stages in transgenic AD mouse models.^81-83^ Future longitudinal studies should provide more insights on the individual neurophysiologial trajectories and their relation with excitation-inhibition imbalance.

### Functional connectivity increases in fronto-central ‘non-hubs’

While mutation carriers did not (yet) show global differences in theta band PLI, they collectively exhibited a degree-dependent shift, involving both higher connectivity in low degree, non-hub regions including inferior orbitofrontal and pre-central cortices and disrupted connectivity in temporoparietal hub regions. This indicates a potential advantage of the HDI over global analyses to capture early connectivity differences at an individual level. Previous literature reproducibly reported global higher theta band phase based connectivity (PLI) in symptomatic sporadic Alzheimer’s disease patients, as well as a disconnection in hub regions (in various frequency bands).^12,16,53,84-86^ There also seems to be a shift in alpha band connectivity from posterior to more anterior regions in later clinical stages of sporadic Alzheimer’s disease.^86^ These findings indicate that a degree-dependent connectivity change as indicated by the HDI may act as early marker for functional network impairment in Alzheimer’s disease.

Orbitofrontal regions present early amyloid accumulation^76^ and showed a similar increase in theta band functional connectivity in symptomatic Alzheimer’s disease patients,^53,84,87,88^ possibly pointing towards an amyloid-driven increase in connectivity.^27^ However, amyloid accumulation may not account for increased theta band PLI in the (pre)central regions, part of the primary motor region, which remain relatively free of pathology until late in the disease process.^89^ Nevertheless, the motor cortex has been involved in higher-order cognitive functions like working memory,^90^ is connected to other brain regions like the prefrontal cortex,^91^ and showed significant hyperexcitability in Alzheimer’s disease patients as indicated with transcranial magnetic stimulation.^92^ This suggests that local amyloid-driven hyperexcitability may elicit functional connectivity changes that extend to other brain regions already in a pre-clinical disease stage.^89^

### Parieto-temporal hub disconnection before the onset of cognitive decline in mutation carriers

A majority of studies reported a comparable decrease in functional connectivity in high (alpha-beta) frequencies, particularly in parieto-temporal regions, in sporadic pre-clinical and symptomatic Alzheimer’s disease patients, using similar robust AECc and other measures.^12,43,53,84,86-88,93,94^ While this has not been investigated in pre-symptomatic mutation carriers before using robust measures, resting-state fMRI studies similarly indicated disrupted functional connectivity, specifically in posterior parts of the default mode network (including the precuneus and posterior cingulate cortex) already at 12 years before expected symptom onset.^95-100^ This fMRI-based hub disconnection was associated with reduced glucose metabolism, increased regional tau deposition at early disease stages, and increased dementia severity.^76,95,97,101^ The specific hub vulnerability may be explained by the high neuronal activity in these hubs, which is metabolically demanding and leads to oxidative stress, excitotoxicity and ultimately, functional impairments and neurodegeneration.^9-11,13,15^ The activity dependent degeneration is likely amplified by the preferential accumulation of amyloid in highly active hub regions, creating a vicious cycle.^102-105^ Together, these findings suggest posterior hub functional disconnections start from a pre-clinical stage and evolves across the Alzheimer’s disease continuum.

Nonetheless, a number of resting-state neurophysiology studies found an increase in the higher alpha frequency band functional connectivity in individuals with Alzheimer’s disease dementia, progressive MCI and cognitively healthy individuals at risk of developing sporadic Alzheimer’s disease, varying across both anterior and posterior regions.^44,52,106-109^ An increased (directed) functional connectivity has also been previously reported in the precuneus, a hub region, during a task in pre-symptomatic mutation carriers at a mean age of 28 years.^52^ A longitudinal resting-state fMRI study in pre-clinical sporadic Alzheimer’s disease patients similarly reported an initial functional connectivity increase before hub disconnection, specifically when amyloid biomarkers become abnormal for the first time.^89^ An early increase in connectivity may be a possible indicator for increased neuronal excitation.^1,17^ As investigated using a computational model, applying a simple activity dependent degeneration rule (lowering synaptic coupling strength as a function of excitatory neuronal firing rate) simulated a biphasic change with initially increased excitatory neuronal firing and functional connectivity before disconnection, particularly affecting hub regions.^17^ The results of the present study, however, do not align with these findings.

A possible explanation for the observed disrupted rather than increased alpha band connectivity in our cohort of pre-clinical mutation carriers would include methodological differences, such as the used connectivity measure and regions of interest, and the relatively late pre-clinical stage of the mutation carriers. At this stage, hub regions may already present prolonged amyloid-driven excitotoxicity causing functional impairments and/or early tau pathology, known to accumulate in the precuneus and posterior cingulate cortex in mutation carriers close to symptom onset and possibly contributing to neuronal silencing.^26,26,101,110-114^ However, a similar scenario would apply to progressive MCI patients, for whom increased alpha band connectivity has also been reported. Alternatively, network hyperexcitability could directly elicit reduced connectivity, preferentially in hub regions, possibly without the need of prolonged hyperexcitability-induced structural alterations, as suggested previously by computational modelling^27,33^ and empirical studies.^28,29^ This could even occur in the presence of early tau pathology, which has recently also been associated with neuronal hyperexcitability.^114-117^ Taken together, it seems that hub disconnection alongside a non-hub hyperconnectivity could point towards increased neuronal excitation in cognitively unimpaired mutation carriers, but more specific measures of excitation-inhibition imbalance like the aperiodic exponent should provide additional evidence.^118,119^

### Neurophysiological changes precede clinical abnormalities

The present study is one of the first to reveal MEG-based abnormalities across the whole brain within cognitively healthy subjects who carry rare pathogenic Alzheimer’s disease mutations. Through comparisons with a well-matched control group, thorough data cleaning, and using robust functional connectivity measures adjusted for volume conduction,^53^ we could accurately characterize whole-brain and region-specific functional brain changes related to mutations in the *APP* and *PSEN1* genes already before cognitive decline. The HDI analyses proved valuable in characterizing and understanding regional connectivity differences at an early disease stage while limiting the multiple comparison problem. These findings highlight the importance of utilizing neurophysiological metrics as a direct and immediate way to quantify abnormal brain function specific for Alzheimer’s disease before the onset of progressive cognitive decline.

### Methodological considerations

The sample size was small because of the rare occurrence of *APP* duplication and *PSEN1* mutations and the additional specific inclusion criteria (e.g. known genetic mutation status and absence of dementia). Due to this low sample size, the findings are explorative in nature. Despite the known differences in various disease characteristics,^120^ we combined *APP* duplication and *PSEN1* mutations to increase statistical power. Prior studies reported similar functional connectivity changes,^97^ but familial and sporadic Alzheimer’s disease patients also present (clinical and pathological) differences^121-123^ and the potential translatability of the present MEG findings requires further investigation. Even though extensive neuropsychological testing showed that the mutation carriers were cognitively healthy, the considerable variability in estimated time to symptom onset may have influenced the results. Notably, some mutation carriers already passed their estimated age of onset by more than 5 years, suggesting that these subjects have a high resilience against Alzheimer’s disease-related pathology, for instance due to a large cognitive reserve attributed to a relatively high level of education^124^ or protective genes.^125^ Additionally, the lack of amyloid and tau biomarker status determination close to MEG registration prevented direct correlation of functional brain network abnormalities to protein aggregates. While using the anatomical AAL brain atlas allows direct comparison of findings between subjects and studies, a more fine-grained parcellation of the brain according to functional and anatomical connection patterns may better represent regional activation or connectivity. Finally, we did not examine the occurrence of (subclinical) epileptiform discharges in our cohort of cognitively unimpaired mutation carriers to confirm network hyperexcitability, because its accurate detection requires long-term (overnight) recordings and suffers from limited visibility and interrater variability.^126-128^

### Future directions

Future studies could consider examining neurophysiological activity using multiple complementary measures that inform about the excitation-inhibition balance from MEG, such as measures integrating various temporal scales, including the aperiodic exponent, pathological oscillatory slowing index or phase-amplitude coupling,^119,129-132^ and/or spatial scales, such as the joint permutation entropy.^30,33,46^ Conducting longitudinal MEG registrations and determining amyloid and tau levels in cerebrospinal fluid or blood could provide enhanced insights into the within-subject neurophysiological trajectory and its association with AD pathology. Neurophysiological recordings during other behavioural states, such as during sleep, a cognitive task or non-invasive stimulation and a more fine-grained parcellation according to integrated multiscale functional and anatomical data (like the Brainnetome atlas^133^) may prove more sensitive to capture early, subtle and regional, neuronal excitation-inhibition balance in Alzheimer’s disease.^128,134-136^ Furthermore, we could employ the activity-dependent degeneration or related computational models of Alzheimer’s disease^17,46,137^ to investigate whether functional connectivity, alongside other disease characteristics, estimates the underlying excitation-inhibition imbalance at an individual level and predicts time to symptom onset or disease progression.

## Conclusions

To conclude, this study provides evidence that neurophysiological alterations occur before cognitive impairment in individuals with autosomal dominant mutations leading to early-onset Alzheimer’s disease. The alterations largely mirror those observed in clinical stages of Alzheimer’s disease and comprise widespread oscillatory slowing and differences in functional connectivity, with a preference in certain brain regions, especially hub regions. Considering previous literature in more advanced stages of sporadic Alzheimer’s disease, this finding does not support a biphasic pattern, but rather points towards a gradual change in neurophysiological activity starting from a pre-clinical and persisting into more advanced stages of the disease. The neurophysiological profile could fit with an underlying hyperexcitable neuronal network, but future studies are required to firmly establish its underlying mechanisms and ultimately manage the growing impact of Alzheimer’s disease on society.

## Supplementary Material

fcae423_Supplementary_Data

## Data Availability

Pseudo-anonymized data of participants who have provided informed consent for data sharing and in-house developed scripts for MEG pre-processing and analyses are available upon formal collaboration agreement. The BrainWave software, written by C. J. Stam, is freely available from https://github.com/CornelisStam/BrainWave.
