# Supplementary material for: Functional network disruption in cognitively unimpaired autosomal dominant Alzheimer’s disease: a magnetoencephalography study
Source: Brain Commun. 2024 Nov 25;6(6):fcae423. doi: 10.1093/braincomms/fcae423 (PMC11660908; doi:10.1093/braincomms/fcae423)
Supplement: fcae423_Supplementary_Data [file fcae423_supplementary_data.pdf]

Supplementary Methods

Whole-exome sequencing

In short, whole exome sequencing data from Amsterdam UMC is generated by the Agilent v4 or v6 kits (~58Mb target region). Sequencing was done on Illumina NovaSeq 6000 (2x150basepair reads) and samples had at least 8Gb raw data per sample. After best practice quality control, genetic variants in the exons and flanking regions (+/- 6 basepairs) from 54 genes associated with monogenic dementia are extracted (listed below). Variants are annotated for pathogenicity in the Alissa Interpret® software (Agilent Technologies - v5.4.0). Variant classification followed the guidelines published by the American College of Medical Genetics and Genomics and the Association for Molecular Pathology [1]. The classification is based on the level of evidence available for each variant. Intronic variants were assessed using webtool SpliceAI (https://spliceailookup.broadinstitute.org/). Only variants with population frequencies <1% were considered. A clinical molecular geneticist interprets the variants for pathogenicity. *C9ORF72* repeat lengths were determined with a commercial kit (AmplideX PCR/CE *C9ORF72* Kit, Asuragen). Variants reported to patients (and in this paper) are only class IV (likely pathogenic) and class V (pathogenic).

Genes associated with monogenic dementia: Hexanucleotide *C9ORF72* repeat, *ALS2* (NM_020919.3), *ANG* (NM_001145.4), *APOE* (NM_001302688.1), *APP* (NM_000484.3), duplications of *APP* (NM_000484.3), *ATP7B* (NM_000053.3), *C19ORF12* (NM_001031726.3), *C9ORF72* (NM_001256054.2), *CHCHD10* (NM_001301339.1), *CHMP2B* (NM_014043.3), *CLN3* (NM_001042432.1), *CLN5* (NM_006493.2), CP (NM_000096.3), *CSF1R* (NM_005211.3), *CTSD* (NM_001909.4), *CTSF* (NM_003793.3), *EIF4G1* (NM_182917.4), *ERBB4* (NM_005235.2), *FUS* (NM_004960.3), *GRN* (NM_002087.3), *HNRNPA1* (NM_031157.3), *HNRNPA2B1* (NM_031243.2), *HTRA1* (NM_002775.4), *ITM2B* (NM_021999.4), *MAPT* (NM_005910.5), *NOTCH3* (NM_000435.2), *NPC1* (NM_000271.4), *NPC2* (NM_006432.3), *OPTN* (NM_001008211.1), *PDGFB* (NM_002608.3), *PDGFRB* (NM_002609.3), *PPT1* (NM_000310.3), *PRKAR1B* (NM_001164761.1), *PRNP* (NM_000311.3), *PSEN1* (NM_000021.3), *PSEN2* (NM_000447.2), *PSENEN* (NM_172341.3), *SERPINI1* (NM_005025.4), *SETX* (NM_015046.5), *SIGMAR1* (NM_005866.3), *SLC20A2* (NM_001257180.1), *SNCA* (NM_000345.3), *SNCB* (NM_001001502.2), *SOD1* (NM_000454.4), *SORL1*

(NM_003105.5), *SPG11* (NM_025137.3), *SQSTM1* (NM_003900.4), *TARDBP* (NM_007375.3), *TBK1* (NM_013254.3), *TREM2* (NM_018965.3), *TYROBP* (NM_003332.3), *UBQLN2* (NM_013444.3), *VCP* (NM_007126.3), *VPS13A* (NM_033305.2), *XPR1* (NM_004736.3).

Cognitive examination and clinical questionnaires

Cognitive function of mutation carriers was assessed via the MMSE and an extensive neuropsychological test battery. To determine if mutation carriers have any self-reported or informant-based cognitive complaints, they completed the Subjective Cognitive Functioning (SCF) questionnaire [2], the Cognitive Change Index (CCI) [3], and a short version of the Amsterdam questionnaire measuring "instrumental activities of daily living (iADL)" in (early) dementia [4] (Supplementary Material). The Hospital Anxiety and Depression Scale (HADS) [5] was also obtained to take into account potential interference of a major anxiety or depressive disorders with cognitive function.

Neuropsychological examination

The MMSE was used as a global index of cognitive performance.[6] The following cognitive domains were evaluated: memory (Visual Association Test, Dutch version of Rey Auditory Verbal Learning Test, Rey Complex Figure Recall Task), attention (Digit span forward, Trail Making Test (TMT) A, Stroop color word test I and II), executive functioning (TMT-B, Digit span backwards, Stroop color word test III, letter fluency test (version D-A-T)), language (category animal fluency, visual association test – 'naming') and visuo-spatial functioning (Rey Complex Figure Copy Task, Visual Object and Space Perception Battery (VOSP) (number location)). Raw test scores were adjusted for age, sex and education and converted to *t*-scores or percentiles.[7] A *t*-score of 36 means that the subject's score is 1.4 standard deviation below the expected score and that 9% of the people with similar age, sex and education performed worse than the studied subject. *T*-scores of 36 or lower were considered abnormal.

For two mutation carriers neuropsychological data was available from the SCIENCe study cohort (2014.019) of the Amsterdam UMC, location VUmc, except for The Rey Complex Figure Tasks, the Digit span forward and backwards and the VOSP. The cognitive domain scores were computed using the available task scores. Ten mutation carriers received cognitive and clinical examination within 2 months before or after MEG recording. One

mutation carrier had clinical and neuropsychological data available from 14 months prior to MEG recording through the SCIENCe study.

Subjective cognitive assessment

Self-reported cognitive decline of mutation carriers was assessed through two questionnaires. Subjective cognitive function compared to 1 and 5 years earlier was assessed with the Subjective Cognitive Functioning (SCF) questionnaire[2] and the Dutch translation of the Cognitive Change Index (CCI),[3] respectively. Psychiatric symptoms were evaluated using the Hospital Anxiety and Depression Scale (HADS).[5] An informant of each participant completed a short version of the Amsterdam questionnaire measuring "instrumental activities of daily living" (IADL) in (early) dementia[4]. The SCF questionnaire for informants was also obtained to assess informant reported decline over a 1-year period.

The SCF questionnaire consists of 4 questions (range -12 to +12).[2] SCF scores <0 represent self or informant reported cognitive decline. The CCI consists of 20 questions (range 20-100).[3] Higher CCI scores reflect worse cognitive function (> 40: mild changes). The HADS is subdivided in 7 items about depression and 7 items about anxiety (range 0 – 21 for each).[5] Higher (sub)scores reflect more psychiatric complaints (0-7: no anxiety or depressive symptoms, 8-10: possible anxiety or depression, 11-21: suspected anxiety disorder or depression). The IADL in (early) dementia was converted to *t*-scores using the Item Response Theory [8] and measures in a range of 20-80. Lower total IADL scores reflect more severe functional impairments and scores below 51.4 were considered abnormal [9].

MRI

For each mutation carrier a standard MRI protocol included a sagittal 3D heavily T1-weighted gradient-echo sequence with coronal reformats, a sagittal 3D T2-weighted fluid-attenuated inversion recovery (FLAIR) turbo/fast spin-echo with axial reformats, a transverse T2-weighted turbo/fast spin-echo, a transverse T2* susceptibility sequence, and diffusion weighted imaging/EPI. All sequences were performed with whole brain coverage [10]. In addition, the protocol was extended with diffusion tensor imaging (DTI). The MRI of the brain was acquired on one of the 3T whole body MRI systems of the Amsterdam UMC, location VUmc (MR750, GE Medical Systems, Milwaukee, WI, USA; Ingenuity TF PET/MR Philips Medical Systems, Best, The Netherlands; Titan, Toshiba Medical Systems, Japan; Magnetom Vida, Siemens Healthineers Nederland B.V., Den Haag, The Netherlands). An experienced neuroradiologist reviewed all scans and performed a visual rating for atrophy of the medial temporal lobe [11], posterior and global cortical atrophy [12-14], white matter hyperintensities [15], lacunes, and microbleeds [16] for all mutation carriers.

Supplementary Results

Post-hoc analyses

After excluding the carrier-control pair, we found similar group differences for whole-brain measures, with lower p-value and somewhat higher effect sizes (not shown). In particular, we found a significantly decreased whole-brain average alpha 2 power in mutation carriers (Mdn = .092, n = 10) compared to controls (Mdn = .117, n = 10), z = -2.803, p = .005, with a large effect size, r = -0.886. The group-wise HDI for alpha band AECc was also stronger (k = -.249) compared to the main analyses. Because of the reduced number of observations we could not perform permutations tests using 2000 permutations and used 1000 permutations instead. At a regional level, more regions showed significant differences, including alpha 2 power primarily in the frontal occipital, temporal, and cingulate cortex (Supplementary Table 8). After multiple comparison correction, a higher number of regions showed significant differences for both spectral power and functional connectivity analyses, particularly for alpha 2 power, peak frequency and alpha band AECc (Supplementary Table 8).

Correlation analyses revealed comparable additional associations between age and whole-brain beta band AECc within mutation carriers (r = .720, p = .019) and between age and whole-brain alpha band AECc in controls (r = .450, p = .013). After correcting for multiple comparisons, no significant correlations were discovered in mutation carriers or controls.

Supplementary Materials. Functional network disruption in cognitively unimpaired autosomal dominant Alzheimer's disease: a magnetoencephalography study. Van Nifterick *et al.*

Supplementary Tables

Supplementary Table 1. Subdivision of regions of interest (ROIs) according to the automatic anatomical labeling atlas (AAL)[17] and with cortical regions ordered as in [18].

Brain lobe	ROI#	Region name left hemisphere	ROI#	Region name right hemisphere
Frontal	1	Rectus_L	40	Rectus_R
	2	Olfactory_L	41	Olfactory_R
	3	Frontal_Sup_Orb_L	42	Frontal_Sup_Orb_R
	4	Frontal_Med_Orb_L	43	Frontal_Med_Orb_R
	5	Frontal_Mid_Orb_L	44	Frontal_Mid_Orb_R
	6	Frontal_Inf_Orb_L	45	Frontal_Inf_Orb_R
	7	Frontal_Sup_L	46	Frontal_Sup_R
	8	Frontal_Mid_L	47	Frontal_Mid_R
	9	Frontal_Inf_Oper_L	48	Frontal_Inf_Oper_R
	10	Frontal_Inf_Tri_L	49	Frontal_Inf_Tri_R
	11	Frontal_Sup_Medial_L	50	Frontal_Sup_Medial_R
Central	12	Supp_Motor_Area_L	51	Supp_Motor_Area_R
	13	Paracentral_Lobule_L	52	Paracentral_Lobule_R
	14	Precentral_L	53	Precentral_R
	15	Rolandic_Oper_L	54	Rolandic_Oper_R
	16	Postcentral_L	55	Postcentral_R
Parietal	17	Parietal_Sup_L	56	Parietal_Sup_R
	18	Parietal_Inf_L	57	Parietal_Inf_R
	19	SupraMarginal_L	58	SupraMarginal_R
	20	Angular_L	59	Angular_R
	21	Precuneus_L	60	Precuneus_R
Occipital	22	Occipital_Sup_L	61	Occipital_Sup_R
	23	Occipital_Mid_L	62	Occipital_Mid_R
	24	Occipital_Inf_L	63	Occipital_Inf_R
	25	Calcarine_L	64	Calcarine_R
	26	Cuneus_L	65	Cuneus_R
	27	Lingual_L	66	Lingual_R
Temporal	28	Fusiform_L	67	Fusiform_R
	29	Heschl_L	68	Heschl_R
	30	Temporal_Sup_L	69	Temporal_Sup_R
	31	Temporal_Mid_L	70	Temporal_Mid_R
	32	Temporal_Inf_L	71	Temporal_Inf_R
	33	Temporal_Pole_Sup_L	72	Temporal_Pole_Sup_R
	34	Temporal_Pole_Mid_L	73	Temporal_Pole_Mid_R
	35	ParaHippocampal_L	74	ParaHippocampal_R
Cingulum	36	Cingulum_Ant_L	75	Cingulum_Ant_R
	37	Cingulum_Mid_L	76	Cingulum_Mid_R
	38	Cingulum_Post_L	77	Cingulum_Post_R
Insula	39	Insula_L	78	Insula_R
Hippocampus	79	Hippocampus_L	80	Hippocampus_R

Supplementary Materials. Functional network disruption in cognitively unimpaired autosomal dominant Alzheimer's disease: a magnetoencephalography study. Van Nifterick *et al.*

Supplementary Table 2. Neuropsychological, subjective cognitive decline and psychiatric test scores. Each column presents test scores for one *APP* or *PSEN1* mutation carrier. Global cognition is presented by total MMSE score. When applicable, neuropsychological test scores were converted to *t*-scores (RAVL, Rey CF recall, Digit span, TMT, Stroop, Fluency) or percentiles (VAT, Rey CF copy). Raw test scores were shown for VAT naming (max 12) and VOSP number location (max 10). Tests were sorted according to cognitive domain (memory, attention, executive functioning, language, visuospatial functioning). Raw test scores were also shown for additional questionnaires SCF, CCI and HADS. IADL *t*-scores were obtained using the Item Response Theory method. Abnormal test scores are indicated in grey. None of the mutation carriers showed clear abnormal functioning on any of the cognitive domains. One mutation carrier had mild attention problems, which could be explained by the level of anxiety. MMSE: Mini-Mental State Examination; VAT: Visual Association Test; RAVL: Dutch version of Rey Auditory Verbal Learning Test; R/T: *t*-score Recall corrected for Total score; Rey CF: Rey Complex Figure Task; TMT: Trail Making Test; Stroop: Stroop color word test; Fluency: Letter (version D-A-T) and category animal fluency test; VOSP: Visual Object and Space Perception Battery (Nr loc: number location); SCF: Subjective Cognitive Functioning questionnaire; CCI: Cognitvie Change Index; HADS: Hospital Anxiety and Depression Scale; A: Anxiety; D: Depression; IADL: Amsterdam questionnaire of instrumental activities of daily living in (early) dementia; *na*: not available.

**Global**	MMSE		30	30	29	28	30	28	28	27	27	29	30
**Memory**	VAT	Total	20	20	20	20	20	20	20	20	20	20	20
	RAVL	Total	53	58	53	70	56	34	43	70	45	35	41
		Recall	49	62	58	68	62	40	47	64	40	38	46
		R/T	42	57	57	51	60	52	53	45	40	52	56
	Rey CF	Recall	*na*	49	42	*na*	73	65	43	40	60	36	34
**Attention**	Digit span	Forward	*na*	50	61	*na*	54	72	50	27	74	54	58
	TMT	A	39	64	49	40	57	52	45	36	55	64	54
	Stroop	1	36	64	30	57	59	31	33	36	57	46	41
		2	37	55	29	72	42	32	38	30	52	37	52
**Executive functioning**	TMT	B	39	67	44	45	50	50	39	31	36	49	67
	Digit span	Backward	*na*	70	48	*na*	64	61	52	48	67	50	68
	Stroop	3	40	59	36	38	45	53	31	41	58	43	58
	Fluency	DAT	42	62	55	57	55	60	66	55	63	46	25
**Language**	Fluency	Animal	40	58	46	65	65	50	52	42	61	58	39
	VAT	Naming	12	12	12	12	12	12	12	12	12	12	12
**Visuospatial functioning**	Rey CF	Copy	*na*	>16	<1	*na*	>16	>16	>16	>16	>16	>16	>16
	VOSP	Nr location	*na*	7	7	*na*	10	10	10	9	*na*	9	10
**Subjective decline**	SCF	Self	0	-1	-3	0	0	-2	-1	-3	0	0	-3
	CCI	Self	23	32	34	27	20	20	20	45	20	20	22
	CCI	Informant	40	23	23	28	20	21	20	39	20	20	25
**Psychiatric symptoms**	HADS	A	9	10	5	1	9	4	6	15	5	4	12
		D	*na*	5	4	5	0	2	2	7	0	0	9
**Interference**	iADL	Informant	63	70	55	68	69	69	69	53	69	70	68

Supplementary Materials. Functional network disruption in cognitively unimpaired autosomal dominant Alzheimer's disease: a magnetoencephalography study. Van Nifterick *et al.*

Supplementary Table 3. Paired permutation test results for spectral measures. Significant *t*-scores and corresponding uncorrected *p*-values for each region of interest (ROI), per frequency band and peak frequency are reported.

	**ROI name**	**ROI #**	***t*-score**	***p*-value**
Delta power				
	Precentral_L	14	1.423	0.034
	Postcentral_L	16	1.793	0.007
	Occipital_Mid_L	23	1.639	0.030
	Cuneus_L	26	1.420	0.050
	Lingual_L	27	2.372	0.040
	Cingulum_Mid_L	37	0.946	0.045
	Cingulum_Post_L	38	1.507	0.034
	Frontal_Med_Orb_R	43	1.383	0.021
	Supp_Motor_Area_R	51	-1.478	0.018
	Precuneus_R	60	1.282	0.037
	Calcarine_R	64	2.056	0.026
	Cuneus_R	65	2.612	0.014
	Lingual_R	66	1.898	0.023
	Cingulum_Post_R	77	1.643	0.024
Theta power				
	Rectus_L	1	2.342	0.011
	Olfactory_L	2	2.185	0.012
	Frontal_Inf_Orb_L	6	2.301	0.030
	Frontal_Inf_Oper_L	9	3.316	0.019
	**Frontal_Inf_Tri_L**	**10**	**3.427**	**0.003**
	Paracentral_Lobule_L	13	1.261	0.050
	Postcentral_L	16	2.419	0.016
	SupraMarginal_L	19	2.102	0.019
	Precuneus_L	21	1.558	0.017
	Occipital_Sup_L	22	2.261	0.026
	Calcarine_L	25	2.312	0.009
	Cuneus_L	26	2.370	0.008
	**Lingual_L**	**27**	**3.764**	**<.001**
	Fusiform_L	28	2.077	0.028
	Temporal_Sup_L	30	2.540	0.044
	Temporal_Mid_L	31	2.333	0.049
	Temporal_Inf_L	32	2.781	0.007
	Temporal_Pole_Sup_L	33	3.008	0.010
	Temporal_Pole_Mid_L	34	2.597	0.008
	ParaHippocampal_L	35	2.911	0.003
	Cingulum_Ant_L	36	2.294	0.029
	Cingulum_Mid_L	37	2.483	0.014
	Cingulum_Post_L	38	2.367	0.006
	**Insula_L**	**39**	**4.446**	**<.001**
	Rectus_R	40	2.081	0.034
	Olfactory_R	41	2.535	0.012
	Frontal_Inf_Orb_R	45	2.477	0.019
	Frontal_Inf_Oper_R	48	3.077	0.016
	Frontal_Inf_Tri_R	49	2.846	0.007
	Paracentral_Lobule_R	52	2.162	0.009
	Postcentral_R	55	1.821	0.037
	SupraMarginal_R	58	2.166	0.034
	Precuneus_R	60	2.339	0.005
	Occipital_Sup_R	61	1.973	0.048
	Occipital_Mid_R	62	2.106	0.036
	Occipital_Inf_R	63	2.195	0.040
	Calcarine_R	64	2.199	0.017
	Cuneus_R	65	2.768	0.007
	Lingual_R	66	2.146	0.036
	Fusiform_R	67	2.150	0.049
	Heschl_R	68	2.252	0.020
	Temporal_Sup_R	69	2.491	0.008
	Temporal_Mid_R	70	2.572	0.015
	Temporal_Pole_Sup_R	72	2.734	0.007
	Temporal_Pole_Mid_R	73	2.510	0.014
	Cingulum_Mid_R	76	2.179	0.031
	Cingulum_Post_R	77	2.569	0.007
	Insula_R	78	2.760	0.011
	Hippocampus_L	79	2.990	0.006
	Hippocampus_R	80	3.294	<.001
Alpha 1 power				
	Frontal_Med_Orb_R	43	-3.050	<.001
	Supp_Motor_Area_R	51	1.562	0.029
	SupraMarginal_R	58	-1.667	0.035

	Temporal_Sup_R	69	-1.682	0.043
Alpha 2 power				
	Olfactory_L	2	-2.288	0.032
	Frontal_Inf_Orb_L	6	-3.176	0.004
	Frontal_Inf_Oper_L	9	-3.243	0.013
	Precentral_L	14	-1.505	0.015
	Rolandic_Oper_L	15	-3.512	0.010
	Postcentral_L	16	-2.475	0.018
	Angular_L	20	-1.869	0.018
	**Occipital_Sup_L**	**22**	**-4.389**	**<.001**
	Occipital_Mid_L	23	-3.719	<.001
	Occipital_Inf_L	24	-3.152	0.010
	Calcarine_L	25	-2.078	0.004
	Cuneus_L	26	-1.990	0.004
	Lingual_L	27	-3.018	0.000
	Fusiform_L	28	-1.850	0.045
	Temporal_Sup_L	30	-2.766	0.042
	Cingulum_Mid_L	37	-2.299	0.022
	Insula_L	39	-2.750	0.019
	Frontal_Med_Orb_R	43	-3.220	0.035
	Supp_Motor_Area_R	51	-1.805	0.014
	Paracentral_Lobule_R	52	-2.493	0.003
	Occipital_Sup_R	61	-3.303	<.001
	Occipital_Mid_R	62	-3.115	<.001
	Occipital_Inf_R	63	-3.233	<.001
	Calcarine_R	64	-2.920	0.002
	Cuneus_R	65	-3.061	0.001
	Lingual_R	66	-2.553	0.005
	Cingulum_Mid_R	76	-2.429	0.003
Beta power				
	Rectus_L	1	-1.702	0.028
	Frontal_Med_Orb_L	4	-1.491	0.026
	Frontal_Inf_Oper_L	9	-2.310	0.014
	Frontal_Inf_Tri_L	10	-2.426	0.014
	Supp_Motor_Area_L	12	-1.916	<.001
	Precentral_L	14	-2.282	0.004
	Cingulum_Ant_L	36	-1.980	0.013
	Insula_L	39	-1.930	0.003
	Rectus_R	40	-2.134	0.001
	Olfactory_R	41	-1.533	0.024
	Frontal_Sup_Orb_R	42	-2.298	0.012
	Frontal_Med_Orb_R	43	-2.151	<.001
	Frontal_Mid_Orb_R	44	-2.170	0.017
	Frontal_Inf_Orb_R	45	-2.037	0.006
	Frontal_Inf_Oper_R	48	-1.838	0.005
	Frontal_Inf_Tri_R	49	-1.642	0.008
	Postcentral_R	55	-1.198	0.008
	Temporal_Pole_Sup_R	72	-2.555	<.001
	Cingulum_Ant_R	75	-2.115	0.027
	Cingulum_Mid_R	76	-1.195	0.037
	Insula_R	78	-2.683	<.001
Peak Frequency				
	Frontal_Inf_Oper_L	9	-3.628	<.001
	Postcentral_L	16	-2.230	0.037
	Parietal_Sup_L	17	-2.877	0.035
	Occipital_Mid_L	23	-2.051	0.036
	Occipital_Inf_L	24	-2.681	0.045
	Cuneus_L	26	-3.246	<.001
	Lingual_L	27	-2.197	0.050
	Fusiform_L	28	-2.228	0.008
	Temporal_Inf_L	32	-1.990	0.016
	ParaHippocampal_L	35	-2.842	0.011
	Cingulum_Mid_L	37	-2.720	0.013
	Insula_L	39	-2.758	0.004
	Frontal_Sup_Orb_R	42	-2.129	0.032
	Frontal_Mid_Orb_R	44	-2.232	0.037
	Supp_Motor_Area_R	51	-3.671	0.000
	Precuneus_R	60	-2.631	0.032
	Occipital_Sup_R	61	-3.214	0.004
	Occipital_Mid_R	62	-2.204	0.017
	Occipital_Inf_R	63	-2.941	0.004
	Calcarine_R	64	-1.699	0.036
	Cuneus_R	65	-2.063	0.009
	Lingual_R	66	-1.991	0.044
	Cingulum_Mid_R	76	-2.503	0.013

Cingulum_Post_R	77	-1.827	0.043
Hippocampus_L	79	-3.239	0.009
Hippocampus_R	80	-1.625	0.005

Bold values highlight statistically significant results after multiple comparison correction. ROI = region of interest, ROI # = ROI number.

Supplementary Materials. Functional network disruption in cognitively unimpaired autosomal dominant Alzheimer's disease: a magnetoencephalography study. Van Nifterick *et al.*

Supplementary Table 4. Paired permutation test resultrs for functional connectivity. Significant *t*-scores and corresponding uncorrected *p*-values per region of interest (ROI), for each frequency band and peak frequency are reported.

	**ROI name**	**ROI #**	***t*-score**	***p*-values**
PLI theta				
	Rectus_L	1	3.376	0.005
	Olfactory_L	2	3.216	0.004
	Frontal_Inf_Oper_L	9	2.063	0.040
	Occipital_Sup_L	22	2.912	<.001
	Occipital_Mid_L	23	2.888	0.032
	Insula_L	39	2.240	0.026
	Olfactory_R	41	3.785	<.001
	Precentral_R	53	2.342	0.029
	SupraMarginal_R	58	-1.926	0.029
	Fusiform_R	67	2.315	0.005
	Cingulum_Post_R	77	3.549	<.001
AECc alpha				
	Frontal_Mid_Orb_L	5	-2.423	0.010
	**Frontal_Mid_L**	**8**	**-5.068**	**<.001**
	Frontal_Inf_Oper_L	9	-2.577	0.050
	Frontal_Sup_Medial_L	11	-3.152	<.001
	Supp_Motor_Area_L	12	-3.058	0.003
	Paracentral_Lobule_L	13	-3.604	0.002
	Precentral_L	14	-3.775	<.001
	Postcentral_L	16	-3.440	0.001
	Cingulum_Ant_L	36	-2.988	0.010
	Cingulum_Mid_L	37	-2.891	0.024
	Insula_L	39	-2.599	0.039
	Olfactory_R	41	-2.389	0.031
	Frontal_Mid_Orb_R	44	-1.607	0.044
	Frontal_Inf_Orb_R	45	-3.443	0.013
	Frontal_Sup_R	46	-2.921	0.009
	Frontal_Mid_R	47	-1.480	0.032
	Frontal_Inf_Oper_R	48	-2.897	<.001
	**Frontal_Inf_Tri_R**	**49**	**-4.168**	**<.001**
	Frontal_Sup_Medial_R	50	-3.540	0.004
	Supp_Motor_Area_R	51	-2.460	0.029
	Paracentral_Lobule_R	52	-3.725	0.017
	Precentral_R	53	-3.238	<.001
	Postcentral_R	55	-2.893	0.023
	Parietal_Sup_R	56	-2.625	0.011
	Parietal_Inf_R	57	-2.911	0.005
	SupraMarginal_R	58	-2.225	0.044
	Angular_R	59	-2.185	0.009
	Fusiform_R	67	-1.755	0.028
	Heschl_R	68	-1.799	0.029
	Temporal_Pole_Sup_R	72	-1.714	0.043
	ParaHippocampal_R	74	-1.837	0.010
	Cingulum_Ant_R	75	-3.760	0.010
	Cingulum_Mid_R	76	-2.022	0.041
	Cingulum_Post_R	77	-2.588	0.031
	Insula_R	78	-1.947	0.015
	Hippocampus_R	80	-2.236	0.025
AECc beta				
	**Rectus_L**	**1**	**-5.054**	**<.001**
	**Olfactory_L**	**2**	**-3.727**	**0.001**
	**Frontal_Sup_Orb_L**	**3**	**-4.231**	**0.001**
	Frontal_Med_Orb_L	4	-2.661	0.039
	**Frontal_Mid_Orb_L**	**5**	**-4.799**	**<.001**
	**Frontal_Inf_Orb_L**	**6**	**-5.479**	**<.001**
	Frontal_Sup_L	7	-2.349	0.014
	Frontal_Mid_L	8	-3.361	0.016
	**Frontal_Inf_Oper_L**	**9**	**-4.954**	**<.001**
	**Frontal_Inf_Tri_L**	**10**	**-3.745**	**0.005**
	Frontal_Sup_Medial_L	11	-3.156	0.006
	**Supp_Motor_Area_L**	**12**	**-3.976**	**0.001**
	**Paracentral_Lobule_L**	**13**	**-4.304**	**<.001**
	**Precentral_L**	**14**	**-4.101**	**<.001**
	Rolandic_Oper_L	15	-2.523	<.001
	Postcentral_L	16	-2.812	0.005
	Parietal_Sup_L	17	-3.106	<.001
	Parietal_Inf_L	18	-2.268	0.018
	Precuneus_L	21	-3.479	0.002
	Occipital_Inf_L	24	-2.726	0.005

Calcarine_L	25	-3.016	0.011
Cuneus_L	26	-3.192	0.009
Lingual_L	27	-3.379	0.003
Temporal_Mid_L	31	-2.715	0.020
Temporal_Inf_L	32	-2.615	0.031
Temporal_Pole_Sup_L	33	-3.213	0.003
Temporal_Pole_Mid_L	34	-2.810	0.045
**Cingulum_Ant_L**	**36**	**-5.679**	**<.001**
**Cingulum_Mid_L**	**37**	**-4.837**	**<.001**
**Cingulum_Post_L**	**38**	**-5.142**	**<.001**
Insula_L	39	-2.963	0.002
**Rectus_R**	**40**	**-5.306**	**<.001**
**Olfactory_R**	**41**	**-4.026**	**0.005**
Frontal_Sup_Orb_R	42	-3.579	0.004
Frontal_Mid_Orb_R	44	-2.376	0.016
Frontal_Inf_Orb_R	45	-3.239	0.007
**Frontal_Sup_R**	**46**	**-4.105**	**0.003**
Frontal_Mid_R	47	-2.353	0.018
Frontal_Inf_Oper_R	48	-2.330	0.016
Frontal_Inf_Tri_R	49	-2.513	0.012
**Frontal_Sup_Medial_R**	**50**	**-4.150**	**0.001**
**Supp_Motor_Area_R**	**51**	**-4.603**	**<.001**
**Paracentral_Lobule_R**	**52**	**-3.999**	**<.001**
Precentral_R	53	-1.927	0.035
Rolandic_Oper_R	54	-1.987	0.035
Postcentral_R	55	-2.580	0.005
Parietal_Sup_R	56	-3.650	0.001
Parietal_Inf_R	57	-2.352	0.036
SupraMarginal_R	58	-2.379	0.027
Angular_R	59	-2.339	0.037
**Precuneus_R**	**60**	**-5.513**	**<.001**
Occipital_Sup_R	61	-2.934	<.001
Occipital_Mid_R	62	-1.937	0.036
Cuneus_R	65	-3.318	<.001
Fusiform_R	67	-2.673	0.017
Heschl_R	68	-2.592	0.003
Temporal_Sup_R	69	-2.715	0.014
Temporal_Inf_R	71	-2.122	0.043
Temporal_Pole_Sup_R	72	-2.756	0.002
Temporal_Pole_Mid_R	73	-2.882	0.009
ParaHippocampal_R	74	-2.606	0.017
**Cingulum_Ant_R**	**75**	**-4.271**	**0.001**
**Cingulum_Mid_R**	**76**	**-4.483**	**<.001**
**Cingulum_Post_R**	**77**	**-4.417**	**<.001**
Insula_R	78	-2.553	0.012
Hippocampus_R	80	-2.663	0.009

Bold values highlight statistically significant results after multiple comparison correction. ROI = region of interest, ROI # = ROI number.

Supplementary Materials. Functional network disruption in cognitively unimpaired autosomal dominant Alzheimer's disease: a magnetoencephalography study. Van Nifterick *et al.*

Supplementary Table 5. Sorted average (weighted) functional degree per region of interest within the control group. Mean and standard deviation (SD) functional degree per region of interest across all control subjects. Results are sorted based on ascending functional degree, thereby highlighting regions characterized as having the highest degree (referred to as hubs) at the top rows, and regions characterized by a lower degree (recognized as non-hubs) at the bottom rows.

	ROI name	ROI #	Mean	SD
PLI theta				
	Temporal_Mid_L	31	0.0981	0.0031
	Temporal_Pole_Mid_L	34	0.0979	0.0032
	Angular_L	20	0.0978	0.0036
	Temporal_Sup_R	69	0.0977	0.0035
	Temporal_Inf_L	32	0.0976	0.0039
	Temporal_Pole_Sup_R	72	0.0973	0.004
	Temporal_Pole_Mid_R	73	0.0971	0.0038
	Frontal_Inf_Oper_R	48	0.0971	0.0029
	Parietal_Inf_R	57	0.0969	0.0034
	Temporal_Pole_Sup_L	33	0.0969	0.0034
	Angular_R	59	0.0969	0.0036
	ParaHippocampal_L	35	0.0968	0.0059
	Rolandic_Oper_L	15	0.0968	0.0024
	Rolandic_Oper_R	54	0.0967	0.0024
	Cingulum_Post_L	38	0.0967	0.0025
	Postcentral_L	16	0.0967	0.0041
	Cuneus_L	26	0.0967	0.0019
	Occipital_Sup_R	61	0.0967	0.0025
	Parietal_Sup_L	17	0.0966	0.0024
	ParaHippocampal_R	74	0.0965	0.0045
	Insula_R	78	0.0965	0.0025
	Cuneus_R	65	0.0965	0.0036
	Paracentral_Lobule_R	52	0.0965	0.0025
	Hippocampus_L	79	0.0964	0.0041
	SupraMarginal_L	19	0.0964	0.0047
	Occipital_Inf_L	24	0.0964	0.0025
	Cingulum_Mid_L	37	0.0964	0.0028
	Frontal_Mid_Orb_L	5	0.0963	0.0034
	Lingual_R	66	0.0963	0.0029
	Precuneus_L	21	0.0963	0.0034
	Frontal_Sup_Medial_R	50	0.0962	0.0035
	Postcentral_R	55	0.0962	0.0029
	Frontal_Inf_Tri_R	49	0.0962	0.0034
	Heschl_R	68	0.0962	0.0036
	Occipital_Sup_L	22	0.0961	0.0033
	Calcarine_L	25	0.0961	0.0032
	Hippocampus_R	80	0.0961	0.0036
	Olfactory_L	2	0.0961	0.003
	Temporal_Inf_R	71	0.096	0.0053
	Fusiform_R	67	0.096	0.0039
	Rectus_R	40	0.096	0.0035
	Temporal_Sup_L	30	0.096	0.0052
	Frontal_Mid_R	47	0.096	0.0028
	Fusiform_L	28	0.096	0.0047
	Precentral_R	53	0.0959	0.0032
	Frontal_Inf_Tri_L	10	0.0959	0.0027
	Rectus_L	1	0.0958	0.0023
	Lingual_L	27	0.0958	0.0034
	Frontal_Mid_Orb_R	44	0.0957	0.0024
	Olfactory_R	41	0.0957	0.0029
	Heschl_L	29	0.0957	0.0046
	Frontal_Inf_Orb_R	45	0.0955	0.0024
	Frontal_Med_Orb_R	43	0.0955	0.0038
	SupraMarginal_R	58	0.0955	0.0029
	Cingulum_Ant_R	75	0.0955	0.003
	Frontal_Sup_Orb_L	3	0.0955	0.0029
	Cingulum_Mid_R	76	0.0955	0.0023
	Temporal_Mid_R	70	0.0954	0.0029
	Frontal_Sup_R	46	0.0954	0.0029
	Frontal_Sup_Medial_L	11	0.0954	0.0028
	Supp_Motor_Area_R	51	0.0953	0.0036
	Parietal_Sup_R	56	0.0953	0.0031
	Parietal_Inf_L	18	0.0953	0.0033
	Frontal_Med_Orb_L	4	0.0953	0.0022
	Precentral_L	14	0.0952	0.0037
	Frontal_Sup_L	7	0.0952	0.0029
	Frontal_Sup_Orb_R	42	0.0952	0.0033

	Cingulum_Post_R	77	0.0951	0.0036
	Precuneus_R	60	0.095	0.0029
	Paracentral_Lobule_L	13	0.095	0.0017
	Frontal_Mid_L	8	0.0948	0.0024
	Calcarine_R	64	0.0948	0.0035
	Occipital_Inf_R	63	0.0948	0.003
	Occipital_Mid_R	62	0.0947	0.0032
	Frontal_Inf_Oper_L	9	0.0946	0.0023
	Cingulum_Ant_L	36	0.0946	0.0028
	Insula_L	39	0.0942	0.0036
	Frontal_Inf_Orb_L	6	0.0942	0.0036
	Supp_Motor_Area_L	12	0.094	0.0024
	Occipital_Mid_L	23	0.0935	0.0027
AECc alpha				
	Fusiform_R	67	0.5326	0.0137
	Hippocampus_R	80	0.5326	0.0112
	Angular_R	59	0.5326	0.0098
	Angular_L	20	0.5325	0.0111
	Hippocampus_L	79	0.5324	0.0119
	ParaHippocampal_R	74	0.5323	0.012
	Fusiform_L	28	0.5318	0.0128
	Temporal_Inf_R	71	0.5317	0.0122
	Parietal_Sup_L	17	0.5314	0.0101
	Temporal_Sup_R	69	0.531	0.0088
	Temporal_Inf_L	32	0.531	0.011
	Parietal_Inf_R	57	0.5309	0.0105
	Heschl_R	68	0.5308	0.0098
	Occipital_Mid_L	23	0.5308	0.0142
	Cingulum_Post_L	38	0.5307	0.0098
	Heschl_L	29	0.5307	0.0108
	Temporal_Mid_L	31	0.5306	0.0129
	ParaHippocampal_L	35	0.5305	0.0118
	Temporal_Mid_R	70	0.5302	0.0132
	Cingulum_Post_R	77	0.5301	0.0107
	SupraMarginal_L	19	0.5296	0.0106
	Parietal_Inf_L	18	0.5296	0.0104
	Cingulum_Mid_L	37	0.529	0.0101
	Precuneus_L	21	0.5288	0.0102
	Rolandic_Oper_R	54	0.5286	0.0098
	Temporal_Sup_L	30	0.5285	0.0094
	SupraMarginal_R	58	0.5284	0.0127
	Rolandic_Oper_L	15	0.5283	0.0085
	Cuneus_R	65	0.5282	0.0125
	Precuneus_R	60	0.5281	0.0112
	Occipital_Sup_R	61	0.5281	0.0134
	Olfactory_R	41	0.528	0.0097
	Olfactory_L	2	0.5277	0.0107
	Insula_L	39	0.5274	0.0092
	Occipital_Sup_L	22	0.5274	0.0123
	Occipital_Mid_R	62	0.5269	0.0126
	Temporal_Pole_Mid_R	73	0.5269	0.0117
	Insula_R	78	0.5268	0.0093
	Occipital_Inf_L	24	0.5265	0.0127
	Cingulum_Mid_R	76	0.5263	0.0097
	Cuneus_L	26	0.5262	0.0124
	Calcarine_R	64	0.5261	0.0129
	Lingual_L	27	0.526	0.0124
	Temporal_Pole_Sup_R	72	0.5259	0.0091
	Temporal_Pole_Sup_L	33	0.5258	0.009
	Parietal_Sup_R	56	0.5254	0.0098
	Lingual_R	66	0.5254	0.0134
	Calcarine_L	25	0.5254	0.013
	Postcentral_L	16	0.5249	0.0091
	Temporal_Pole_Mid_L	34	0.5241	0.0113
	Rectus_R	40	0.5238	0.0087
	Postcentral_R	55	0.5237	0.0089
	Frontal_Inf_Orb_R	45	0.5237	0.008
	Rectus_L	1	0.5229	0.0095
	Paracentral_Lobule_R	52	0.5226	0.0089
	Frontal_Inf_Oper_L	9	0.5221	0.0087
	Frontal_Inf_Oper_R	48	0.5218	0.0068
	Paracentral_Lobule_L	13	0.5216	0.0079
	Precentral_R	53	0.5214	0.0093
	Occipital_Inf_R	63	0.5206	0.0116
	Precentral_L	14	0.5206	0.0084

	Frontal_Inf_Orb_L	6	0.5201	0.01
	Frontal_Inf_Tri_R	49	0.5198	0.0057
	Supp_Motor_Area_L	12	0.5197	0.0083
	Supp_Motor_Area_R	51	0.5195	0.0086
	Cingulum_Ant_L	36	0.5194	0.0079
	Cingulum_Ant_R	75	0.519	0.0082
	Frontal_Sup_Orb_R	42	0.5188	0.0077
	Frontal_Inf_Tri_L	10	0.5181	0.009
	Frontal_Sup_Orb_L	3	0.5175	0.0089
	Frontal_Med_Orb_R	43	0.5175	0.0068
	Frontal_Sup_R	46	0.5165	0.0075
	Frontal_Mid_Orb_R	44	0.5163	0.0076
	Frontal_Mid_L	8	0.5159	0.0066
	Frontal_Mid_Orb_L	5	0.5158	0.0099
	Frontal_Med_Orb_L	4	0.5157	0.0068
	Frontal_Mid_R	47	0.5145	0.0074
	Frontal_Sup_L	7	0.5144	0.0075
	Frontal_Sup_Medial_L	11	0.5141	0.0075
	Frontal_Sup_Medial_R	50	0.5133	0.007
AECc beta				
	Parietal_Inf_R	57	0.5293	0.0086
	Angular_R	59	0.5287	0.0082
	Parietal_Inf_L	18	0.5279	0.0084
	Angular_L	20	0.5275	0.009
	SupraMarginal_R	58	0.5274	0.0081
	Cingulum_Post_L	38	0.5271	0.0082
	Cingulum_Post_R	77	0.5261	0.0076
	Precuneus_R	60	0.526	0.0071
	Parietal_Sup_L	17	0.5258	0.0068
	Precuneus_L	21	0.5256	0.0068
	SupraMarginal_L	19	0.5251	0.0079
	Heschl_R	68	0.525	0.0068
	Temporal_Mid_R	70	0.5248	0.0076
	Cingulum_Mid_L	37	0.5247	0.008
	Postcentral_R	55	0.5247	0.0066
	Parietal_Sup_R	56	0.5245	0.005
	Temporal_Sup_R	69	0.5243	0.0069
	Hippocampus_R	80	0.5242	0.0067
	Temporal_Inf_R	71	0.524	0.007
	Cingulum_Mid_R	76	0.5239	0.0086
	Hippocampus_L	79	0.5233	0.0077
	Rolandic_Oper_R	54	0.5226	0.0069
	Frontal_Inf_Oper_R	48	0.5225	0.0073
	Precentral_R	53	0.5225	0.0081
	ParaHippocampal_R	74	0.5225	0.0069
	Temporal_Mid_L	31	0.5222	0.0067
	Postcentral_L	16	0.5221	0.0071
	Fusiform_R	67	0.5221	0.0069
	Precentral_L	14	0.5221	0.0078
	Supp_Motor_Area_R	51	0.522	0.009
	Rolandic_Oper_L	15	0.5218	0.0074
	Insula_R	78	0.5216	0.0079
	Heschl_L	29	0.5215	0.0067
	Temporal_Inf_L	32	0.5212	0.0075
	Paracentral_Lobule_R	52	0.5211	0.007
	Fusiform_L	28	0.521	0.0067
	ParaHippocampal_L	35	0.5209	0.0068
	Olfactory_R	41	0.5209	0.0072
	Olfactory_L	2	0.5208	0.0072
	Temporal_Sup_L	30	0.5207	0.007
	Occipital_Sup_R	61	0.5206	0.0058
	Frontal_Inf_Oper_L	9	0.5205	0.0061
	Insula_L	39	0.5203	0.0072
	Occipital_Mid_L	23	0.5203	0.0058
	Supp_Motor_Area_L	12	0.5202	0.008
	Temporal_Pole_Sup_R	72	0.5198	0.0063
	Occipital_Sup_L	22	0.5197	0.0056
	Cuneus_L	26	0.5194	0.0058
	Occipital_Mid_R	62	0.5193	0.0047
	Cuneus_R	65	0.5192	0.0061
	Paracentral_Lobule_L	13	0.5191	0.0067
	Frontal_Inf_Tri_R	49	0.5187	0.0052
	Frontal_Sup_R	46	0.5182	0.0077
	Temporal_Pole_Mid_R	73	0.5182	0.0049
	Rectus_R	40	0.5182	0.0067

Rectus_L	1	0.5178	0.0066
Frontal_Mid_R	47	0.5177	0.0068
Frontal_Inf_Orb_R	45	0.5177	0.0063
Temporal_Pole_Sup_L	33	0.5176	0.006
Frontal_Mid_L	8	0.5174	0.0067
Calcarine_R	64	0.5174	0.0046
Frontal_Inf_Tri_L	10	0.5174	0.0062
Lingual_L	27	0.5173	0.0052
Cingulum_Ant_L	36	0.5169	0.0071
Temporal_Pole_Mid_L	34	0.5168	0.0054
Lingual_R	66	0.5167	0.0053
Frontal_Inf_Orb_L	6	0.5165	0.006
Cingulum_Ant_R	75	0.5161	0.0075
Calcarine_L	25	0.5159	0.0042
Occipital_Inf_L	24	0.5159	0.0062
Frontal_Sup_Orb_R	42	0.5148	0.0064
Frontal_Sup_L	7	0.5147	0.0067
Frontal_Sup_Medial_L	11	0.5141	0.0067
Frontal_Sup_Orb_L	3	0.5138	0.0067
Occipital_Inf_R	63	0.5136	0.0042
Frontal_Sup_Medial_R	50	0.5135	0.0066
Frontal_Mid_Orb_R	44	0.5131	0.0062
Frontal_Mid_Orb_L	5	0.5131	0.0058
Frontal_Med_Orb_R	43	0.5128	0.0067
Frontal_Med_Orb_L	4	0.5121	0.0061

Supplementary Table 6. Hub disruption index results per functional connectivity measure. Results of a simple linear regression analyses to determine the hub disruption index (HDI) for each functional connectivity measure and frequency band. A significant p-value indicates a significant correlation between the regional functional degree in the reference (control) network, and the difference in functional degree of the respective region in a target network (*APP* and *PSEN1* mutation carriers). Group-level HDI was calculated using the average regional functional degree for mutation carriers (N = 11) and controls (N = 33) . Subject-level HDI was calculated using the regional functional degree of one mutation carrier compared to the average regional functional degree of their (surrogate) age-and gender-matched controls (N = 3).

		**HDI**	***Y*-intercept**	**Goodness of Fit (R^2)**	***F*(DFn, DFd)**	***p*-value**
Group-level						
	PLI theta	-0.8603	0.08384	0.1495	13.71 (1,78)	<.001
	AECc alpha	-0.1624	0.07663	0.1497	13.74 (1,78)	<.001
	AECc beta	-0.1498	0.07088	0.1648	15.39 (1,78)	<.001
Subject-level						
	PLI theta					
		-1,098	0,1069	0,2317	23,52 (1,78)	<.001
		-0,6685	0,06349	0,1408	12,78 (1,78)	<.001
		-0,6664	0,06476	0,2369	24,22 (1,78)	<.001
		-0,8496	0,0834	0,1488	13,64 (1,78)	<.001
		-0,8398	0,07941	0,2341	23,84 (1,78)	<.001
		-1,043	0,1022	0,365	44,83 (1,78)	<.001
		-1,008	0,0945	0,3026	33,85 (1,78)	<.001
		-0,9861	0,09695	0,2636	27,92 (1,78)	<.001
		-0,7017	0,07465	0,08384	7,138 (1,78)	.009
		-1,259	0,1201	0,409	53,98 (1,78)	<.001
		-0,8359	0,08278	0,1174	10,37 (1,78)	.002
	AECc alpha					
		0,09229	-0,0323	0,001658	0,1295 (1,78)	.720
		0,309	-0,1724	0,07806	6,604 (1,78)	.012
		-0,6759	0,3419	0,4513	64,16 (1,78)	<.001
		-0,4946	0,249	0,2864	31,3 (1,78)	<.001
		-0,7976	0,4051	0,5114	81,64 (1,78)	<.001
		-0,8687	0,4428	0,6361	136,4 (1,78)	<.001
		-0,5398	0,2933	0,05115	4,205 (1,78)	.044
		-1,078	0,5454	0,6028	118,4 (1,78)	<.001
		-0,8137	0,4143	0,6268	131 (1,78)	<.001
		-0,695	0,3444	0,4528	64,54 (1,78)	<.001
		-0,7761	0,3927	0,4688	68,84 (1,78)	<.001
	AECc beta					
		-0,4121	0,2097	0,1887	18,14 (1,78)	<.001
		0,4808	-0,2478	0,08068	6,846 (1,78)	.011
		-0,6727	0,3401	0,5714	104 (1,78)	<.001
		-0,5481	0,2764	0,3171	36,22 (1,78)	<.001
		-0,7028	0,353	0,8113	335,3 (1,78)	<.001
		-0,1197	0,05631	0,0112	0,8835 (1,78)	0.35
		0,07472	-0,0357	0,00544	0,4266 (1,78)	.516
		-0,5236	0,2612	0,2358	24,07 (1,78)	<.001
		-0,6304	0,3222	0,5247	86,12 (1,78)	<.001
		-0,8369	0,4184	0,6256	130,3 (1,78)	<.001
		-0,6787	0,3371	0,4764	70,98 (1,78)	<.001

Supplementary Materials. Functional network disruption in cognitively unimpaired autosomal dominant Alzheimer's disease: a magnetoencephalography study. Van Nifterick *et al.*

Supplementary Table 7. Correlation analyses using distinct calculation methods for EYBSO.

		global mean				HDI		
		**Theta power**	**Peak frequency**	**AECc alpha**	**AECc beta**	**HDI PLI theta**	**HDI AECc alpha**	**HDI AECc beta**
EYBSO_parent	*r*	.096	-.109	-.506	**-.670**	.018	-.232	**-.624**
	p-value	.780	.749	.113	**.024**	.958	.492	**.040**
	n	11	11	11	11	11	11	11
EYBSO_family	*r*	.309	-.436	**-.627**	-.536	.018	-.373	-.591
	p-value	.355	.180	**.039**	.089	.958	.259	.056
	n	11	11	11	11	11	11	11
EYBSO_mutation	*r*	.091	-.100	-.433	-.487	.196	-.046	-.565
	p-value	.790	.769	.184	.128	.564	.894	.070
	n	11	11	11	11	11	11	11

Bold value highlights statistically significant results ($p < .05$).

Supplementary Materials. Functional network disruption in cognitively unimpaired autosomal dominant Alzheimer's disease: a magnetoencephalography study. Van Nifterick *et al.*

Supplementary Table 8. Post-hoc statistical test results for alpha 2 power, peak frequency and alpha band AECc. Group-wise t-scores and p-values per region of interest (ROI) based on paired permutation tests (1000 permutations), after excluding an outlier.

	ROI_name	ROI #	t-score	*p*-value
Alpha 2				
	**Rectus_L**	**1**	**-5.023**	**<.001**
	**Olfactory_L**	**2**	**-6.817**	**<.001**
	Frontal_Sup_Orb_L	3	-2.574	0.045
	Frontal_Inf_Orb_L	6	-3.508	0.002
	Frontal_Sup_L	7	-2.349	0.038
	**Frontal_Inf_Oper_L**	**9**	**-5.076**	**0.002**
	Supp_Motor_Area_L	12	-2.714	<.001
	Paracentral_Lobule_L	13	-3.328	<.001
	Precentral_L	14	-1.521	0.016
	**Rolandic_Oper_L**	**15**	**-4.844**	**<.001**
	Postcentral_L	16	-2.912	0.008
	Parietal_Sup_L	17	-2.591	0.028
	Parietal_Inf_L	18	-3.708	<.001
	Angular_L	20	-4.355	<.001
	Precuneus_L	21	-2.538	0.014
	**Occipital_Sup_L**	**22**	**-4.786**	**<.001**
	**Occipital_Mid_L**	**23**	**-5.409**	**<.001**
	Occipital_Inf_L	24	-4.193	<.001
	Calcarine_L	25	-2.932	<.001
	Cuneus_L	26	-2.592	<.001
	Lingual_L	27	-3.201	<.001
	**Fusiform_L**	**28**	**-4.696**	**<.001**
	**Heschl_L**	**29**	**-4.645**	**0.020**
	Temporal_Sup_L	30	-4.174	0.010
	Temporal_Mid_L	31	-3.166	0.028
	Temporal_Inf_L	32	-2.887	0.018
	Temporal_Pole_Sup_L	33	-3.250	0.020
	ParaHippocampal_L	35	-3.636	0.002
	Cingulum_Ant_L	36	-3.116	0.012
	**Cingulum_Mid_L**	**37**	**-7.199**	**<.001**
	Cingulum_Post_L	38	-3.325	0.012
	Insula_L	39	-3.705	0.006
	**Rectus_R**	**40**	**-6.715**	**<.001**
	**Olfactory_R**	**41**	**-4.668**	**0.006**
	**Frontal_Sup_Orb_R**	**42**	**-5.597**	**0.004**
	**Frontal_Med_Orb_R**	**43**	**-4.907**	**0.006**
	Frontal_Mid_Orb_R	44	-3.531	0.024
	Frontal_Sup_R	46	-2.844	0.002
	Frontal_Mid_R	47	-2.301	0.004
	Frontal_Inf_Oper_R	48	-3.297	<.001
	Frontal_Inf_Tri_R	49	-1.657	0.002
	Frontal_Sup_Medial_R	50	-3.708	0.010
	Supp_Motor_Area_R	51	-2.595	<.001
	**Paracentral_Lobule_R**	**52**	**-5.268**	**<.001**
	Precentral_R	53	-3.216	<.001
	Rolandic_Oper_R	54	-3.611	<.001
	Postcentral_R	55	-2.205	0.029
	Parietal_Sup_R	56	-2.920	0.010
	Parietal_Inf_R	57	-2.782	0.014
	SupraMarginal_R	58	-3.759	<.001
	Angular_R	59	-3.915	<.001
	Precuneus_R	60	-3.914	<.001
	**Occipital_Sup_R**	**61**	**-5.400**	**<.001**
	**Occipital_Mid_R**	**62**	**-5.430**	**<.001**
	**Occipital_Inf_R**	**63**	**-14.477**	**<.001**
	Calcarine_R	64	-4.192	<.001
	**Cuneus_R**	**65**	**-4.989**	**<.001**
	Lingual_R	66	-3.533	<.001
	Heschl_R	68	-3.331	0.004
	Temporal_Sup_R	69	-2.263	0.050
	ParaHippocampal_R	74	-3.073	0.004
	Cingulum_Ant_R	75	-3.557	0.012
	**Cingulum_Mid_R**	**76**	**-8.769**	**<.001**
	**Cingulum_Post_R**	**77**	**-5.756**	**<.001**
	Hippocampus_L	79	-2.922	0.038
	Hippocampus_R	80	-3.417	0.008
Peak frequency				
	Olfactory_L	2	-1.907	0.036
	**Frontal_Inf_Oper_L**	**9**	**-5.087**	**<.001**

	Precentral_L	14	-1.669	0.046
	Postcentral_L	16	-2.808	0.004
	**Parietal_Sup_L**	**17**	**-4.256**	**0.006**
	Parietal_Inf_L	18	-2.952	0.020
	SupraMarginal_L	19	-2.258	0.020
	Angular_L	20	-2.356	0.030
	Precuneus_L	21	-1.711	0.014
	Occipital_Mid_L	23	-2.150	0.024
	Cuneus_L	26	-3.338	<.001
	Lingual_L	27	-2.351	0.034
	Fusiform_L	28	-3.241	<.001
	Temporal_Mid_L	31	-2.250	0.048
	Temporal_Inf_L	32	-2.187	0.010
	**Temporal_Pole_Sup_L**	**33**	**-5.647**	**<.001**
	ParaHippocampal_L	35	-3.468	<.001
	Cingulum_Mid_L	37	-3.477	0.006
	Insula_L	39	-3.411	<.001
	Olfactory_R	41	-2.582	0.016
	Frontal_Sup_Orb_R	42	-2.277	0.030
	Frontal_Mid_Orb_R	44	-3.167	0.004
	Frontal_Inf_Orb_R	45	-1.925	0.043
	Frontal_Mid_R	47	-1.823	0.035
	Frontal_Inf_Oper_R	48	-3.137	<.001
	Frontal_Inf_Tri_R	49	-2.286	0.012
	**Supp_Motor_Area_R**	**51**	**-4.130**	**<.001**
	Paracentral_Lobule_R	52	-2.613	0.002
	Rolandic_Oper_R	54	-2.044	0.010
	Postcentral_R	55	-2.804	0.021
	Parietal_Sup_R	56	-3.245	0.006
	Angular_R	59	-1.788	<.001
	Precuneus_R	60	-3.148	0.010
	**Occipital_Sup_R**	**61**	**-4.480**	**<.001**
	Occipital_Mid_R	62	-2.552	0.006
	**Occipital_Inf_R**	**63**	**-4.056**	**<.001**
	Calcarine_R	64	-2.139	0.006
	Cuneus_R	65	-2.563	0.002
	Lingual_R	66	-2.233	0.026
	Heschl_R	68	-2.267	0.026
	Temporal_Pole_Mid_R	73	-2.350	0.040
	ParaHippocampal_R	74	-3.210	0.008
	Cingulum_Ant_R	75	-1.735	0.003
	**Cingulum_Mid_R**	**76**	**-4.897**	**<.001**
	Cingulum_Post_R	77	-2.334	0.018
	Insula_R	78	-1.407	0.036
	Hippocampus_L	79	-3.464	0.004
	Hippocampus_R	80	-2.334	<.001
AECc alpha				
	Olfactory_L	2	-2.484	0.024
	Frontal_Mid_Orb_L	5	-2.392	0.018
	Frontal_Sup_L	7	-3.266	<.001
	Frontal_Mid_L	8	-4.607	<.001
	**Frontal_Sup_Medial_L**	**11**	**-6.675**	**<.001**
	**Supp_Motor_Area_L**	**12**	**-6.946**	**<.001**
	**Paracentral_Lobule_L**	**13**	**-9.818**	**<.001**
	Precentral_L	14	-3.410	0.004
	**Postcentral_L**	**16**	**-5.828**	**<.001**
	Parietal_Sup_L	17	-3.654	0.046
	Parietal_Inf_L	18	-2.375	0.030
	Precuneus_L	21	-3.459	0.034
	Occipital_Sup_L	22	-2.714	0.034
	Occipital_Mid_L	23	-2.478	0.050
	Occipital_Inf_L	24	-2.240	0.036
	Cuneus_L	26	-3.519	0.016
	Lingual_L	27	-2.469	0.035
	Temporal_Inf_L	32	-2.552	0.048
	ParaHippocampal_L	35	-2.575	0.024
	Cingulum_Ant_L	36	-4.672	<.001
	**Cingulum_Mid_L**	**37**	**-5.127**	**0.002**
	Cingulum_Post_L	38	-4.556	0.004
	Insula_L	39	-2.878	0.030
	Rectus_R	40	-2.396	0.042
	Olfactory_R	41	-3.801	0.006
	Frontal_Mid_Orb_R	44	-2.027	0.022
	Frontal_Inf_Orb_R	45	-3.845	0.012
	Frontal_Sup_R	46	-3.490	0.014

Frontal_Mid_R	47	-3.534	<.001
Frontal_Inf_Oper_R	48	-3.026	0.002
Frontal_Inf_Tri_R	49	-4.205	<.001
Frontal_Sup_Medial_R	50	-4.622	<.001
Supp_Motor_Area_R	51	-4.032	<.001
**Paracentral_Lobule_R**	**52**	**-9.154**	**<.001**
Precentral_R	53	-3.902	<.001
Rolandic_Oper_R	54	-2.190	0.033
Postcentral_R	55	-3.883	0.010
**Parietal_Sup_R**	**56**	**-5.146**	**<.001**
Parietal_Inf_R	57	-3.783	0.002
SupraMarginal_R	58	-3.366	0.008
Angular_R	59	-3.807	<.001
**Precuneus_R**	**60**	**-5.165**	**0.002**
Occipital_Sup_R	61	-4.031	0.004
Occipital_Mid_R	62	-2.222	0.034
Occipital_Inf_R	63	-1.942	<.001
Calcarine_R	64	-3.279	0.008
Cuneus_R	65	-4.535	0.006
Lingual_R	66	-2.748	0.010
Fusiform_R	67	-2.251	0.008
Heschl_R	68	-2.280	0.014
Temporal_Sup_R	69	-1.957	0.037
Temporal_Pole_Sup_R	72	-2.012	0.022
ParaHippocampal_R	74	-1.941	0.014
**Cingulum_Ant_R**	**75**	**-5.596**	**<.001**
Cingulum_Mid_R	76	-3.937	<.001
Cingulum_Post_R	77	-4.675	0.002
Insula_R	78	-2.087	0.012
Hippocampus_L	79	-2.467	0.026
Hippocampus_R	80	-2.649	0.016

Bold values highlight statistically significant results after multiple comparison correction. ROI = region of interest, ROI # = ROI number.

Supplementary Figures

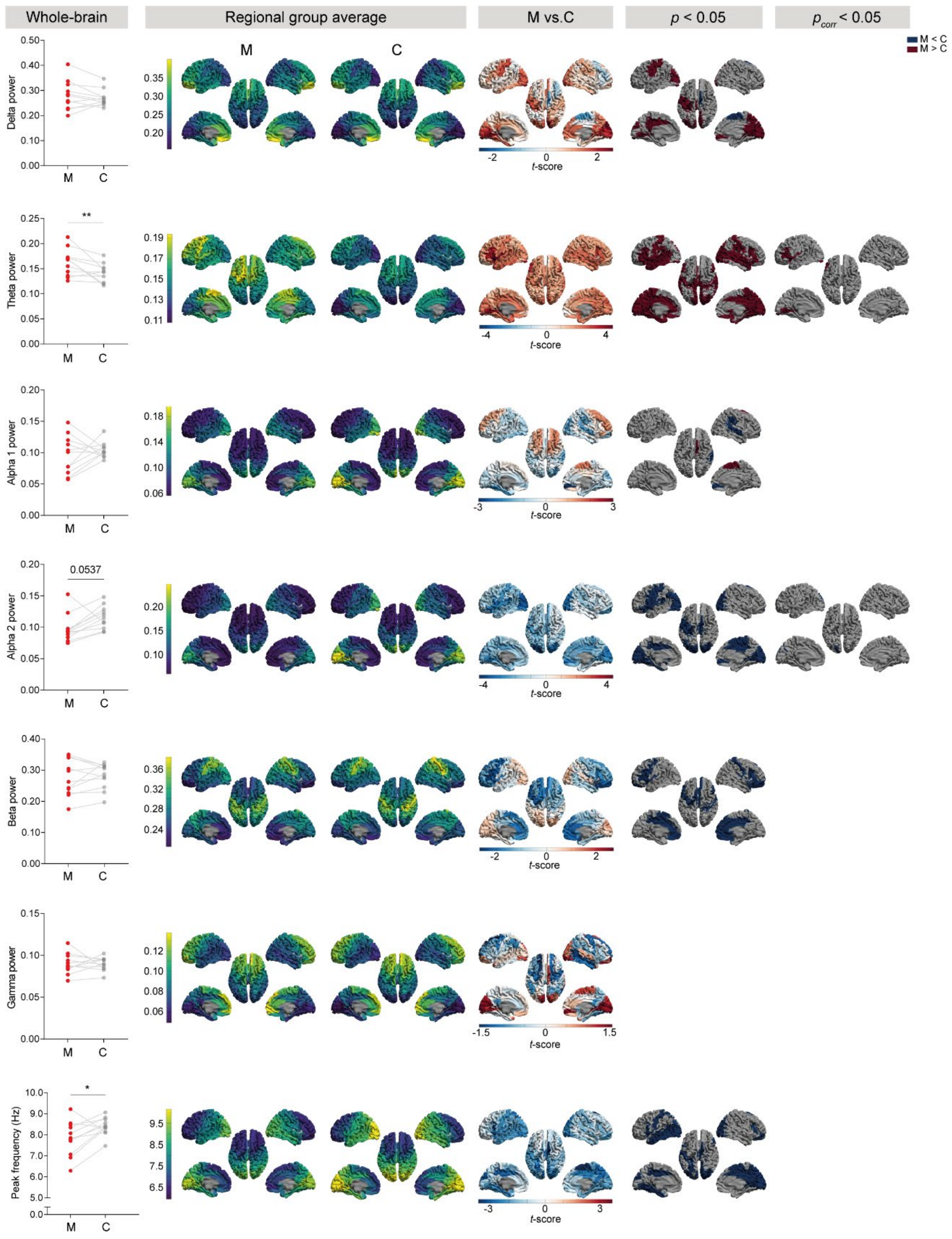

Supplementary Figure 1. **Whole-brain average and regional spectral activity for mutation carriers and controls**. The data points in the graphs in the left column present the whole-brain average MEG-based relative power in each frequency band and peak frequency

for mutation carriers (M, $n = 11$) and (surrogate) age-and gender-matched controls (C, $n = 11$). Wilcoxon matched-pairs signed rank sum test revealed significant differences between mutation carriers and controls. The brain color plots in the right columns indicate from left to right: the regional spectral measures averaged across all mutation carriers ($n = 11$) or (surrogate) controls ($n = 11$), the differences between mutation carriers and (surrogate) controls presented in *t*-scores based on paired permutation tests, the significant *t*-scores ($p < .05$), and the significant *t*-scores after multiple comparisons correction.

Supplementary Materials. Functional network disruption in cognitively unimpaired autosomal dominant Alzheimer's disease: a magnetoencephalography study. Van Nifterick *et al.*

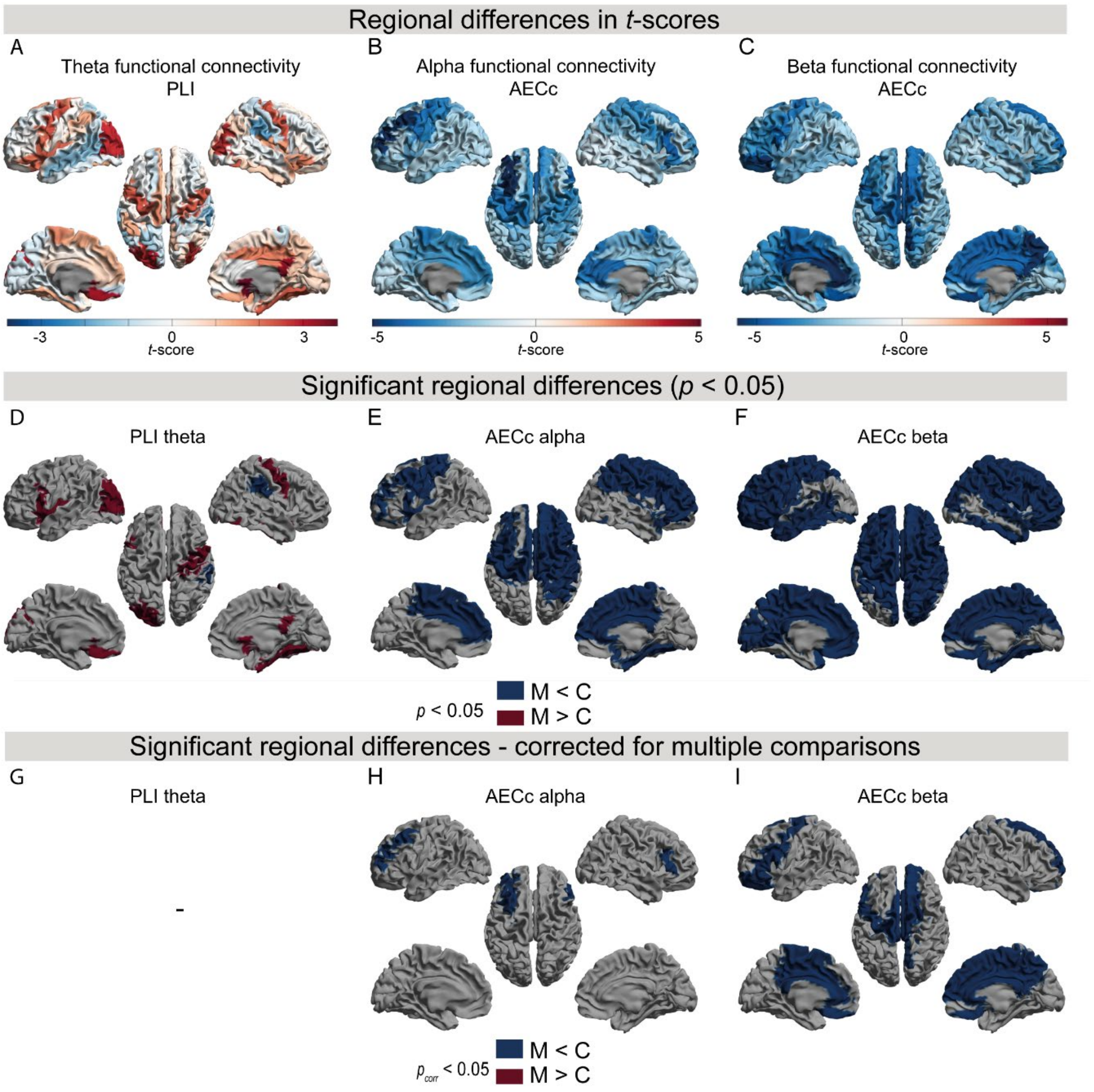

Supplementary Figure 2. Functional connectivity differences between mutation carriers and controls. A-C) The brain color plots depict the regional *t*-scores based on paired permutation tests to assess differences in functional connectivity (theta band PLI (**A**), alpha band AECc (**B**) and beta band AECc (**C**)) between mutation carriers (M, $n = 11$) and (surrogate) controls (C, $n = 11$). **D-F)** Thresholded brain color plots present regions with significant functional connectivity differences between mutation carriers (M, N = 11) and (surrogate) controls (C, N = 11, $p < .05$) using the permutation tests. **G-I)** The thresholded brain color plots present regional significant differences after multiple comparisons correction (using the maximal statistic). No regions showed significant group differences for theta band PLI after multiple comparisons corrections, and, thus, results were not visualized. In- and decreased functional connectivity in mutation carriers compared to controls are indicated in

red and blue, respectively. PLI, phase lag index; AECc, amplitude envelope correlation corrected for volume conduction.

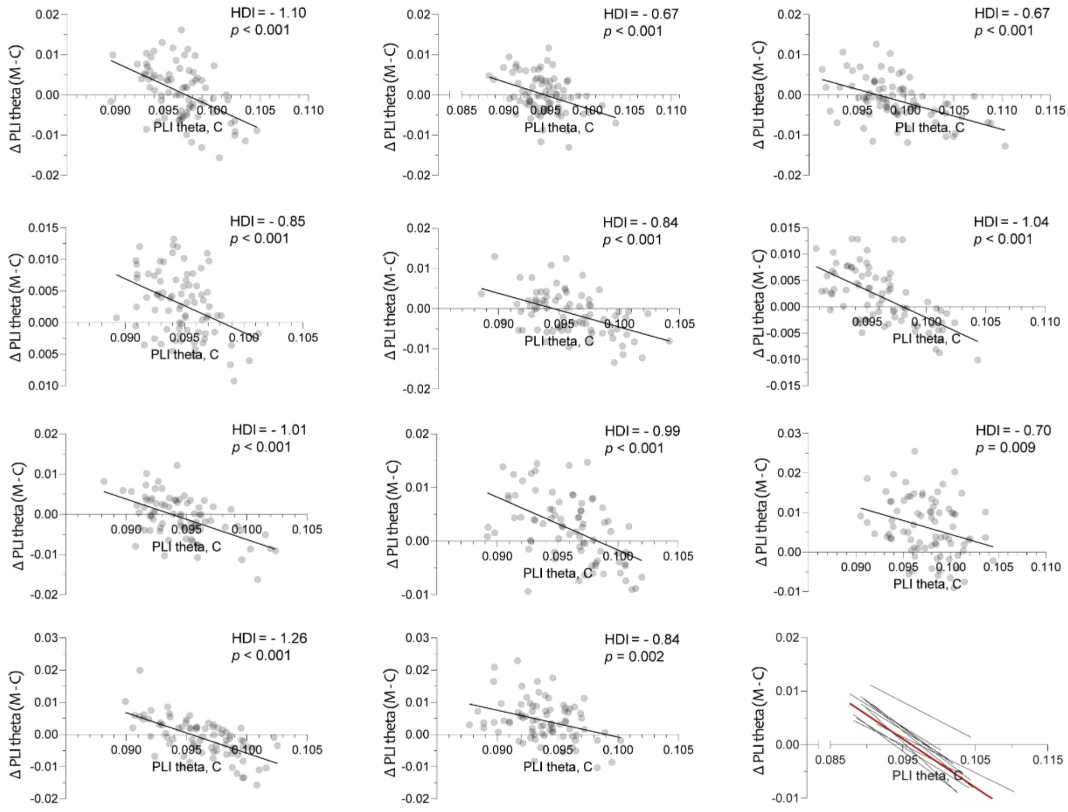

Supplementary Figure 3. Individual theta band PLI hub disruption index for mutation carriers. Each subplot shows the hub disruption index (HDI) results based on theta band PLI for each mutation carrier (*n* = 11). The difference in regional functional degree (for each of the 80 regions of the AAL atlas) between a mutation carrier (M, *n* = 1) and (surrogate) control (C, *n* = 1) is presented on the *y*-axis as function of the regional functional degree in the surrogate control on the *x*-axis. The functional degree was computed as the average connectivity strength (measured with PLI in the theta band) between one region and all other brain regions. The (surrogate) control values were obtained by averaging across three healthy controls that matched to one mutation carrier in respect to age and sex. Simple linear regression analyses was conducted to obtain the HDI for each mutation carrier. The bottom right plot shows the 11 linear regression lines for all mutation carriers. The colored line reflects the linear fit when all data points (80 regions for *n* = 11 mutation carriers and surrogate controls, not shown) are taken into account, indicating the average HDI for mutation carriers. Statistical test details and results are reported in Supplementary Table 6. PLI, phase lag index; AAL, automated anatomical labeling.

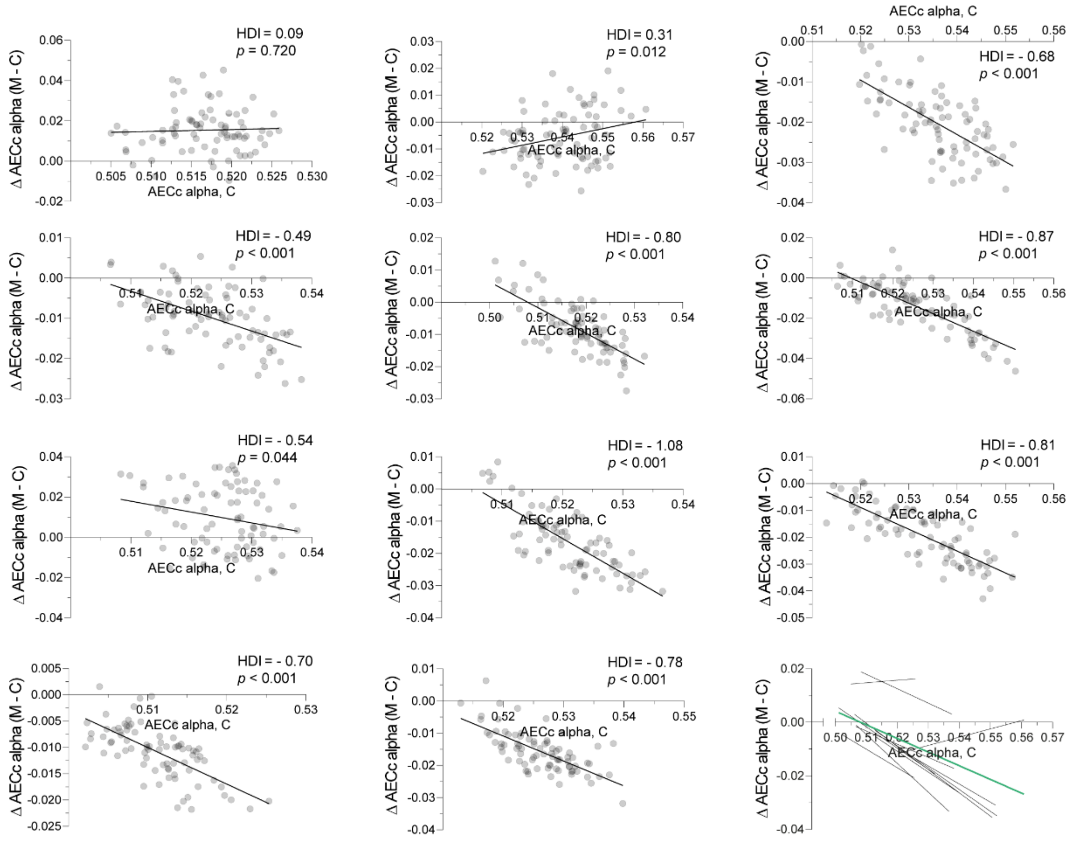

Supplementary Figure 4. **Individual alpha band AECc hub disruption index for mutation carriers.** Each subplot shows the hub disruption index (HDI) results based on alpha band AECc for each mutation carrier (n = 11). The difference in regional functional degree (for each of the 80 regions of the AAL atlas) between a mutation carrier (M, n = 1) and (surrogate) control (C, n = 1) is presented on the y-axis as function of the regional functional degree in the surrogate control on the x-axis. The functional degree was computed as the average connectivity strength (measured with AECc in the alpha band) between one region and all other brain regions. The (surrogate) control values were obtained by averaging across three healthy controls that matched to one mutation carrier in respect to age and sex. Simple linear regression analyses was conducted to obtain the HDI for each mutation carrier. The bottom right plot shows the 11 linear regression lines for all mutation carriers. The colored line reflects the linear fit when all data points (80 regions for n = 11 mutation carriers and surrogate controls, not shown) are taken into account, indicating the average HDI for mutation carriers. Statistical test details and results are reported in Supplementary Table 6. AECc, amplitude envelope correlation corrected for volume conduction; AAL, automated anatomical labeling.

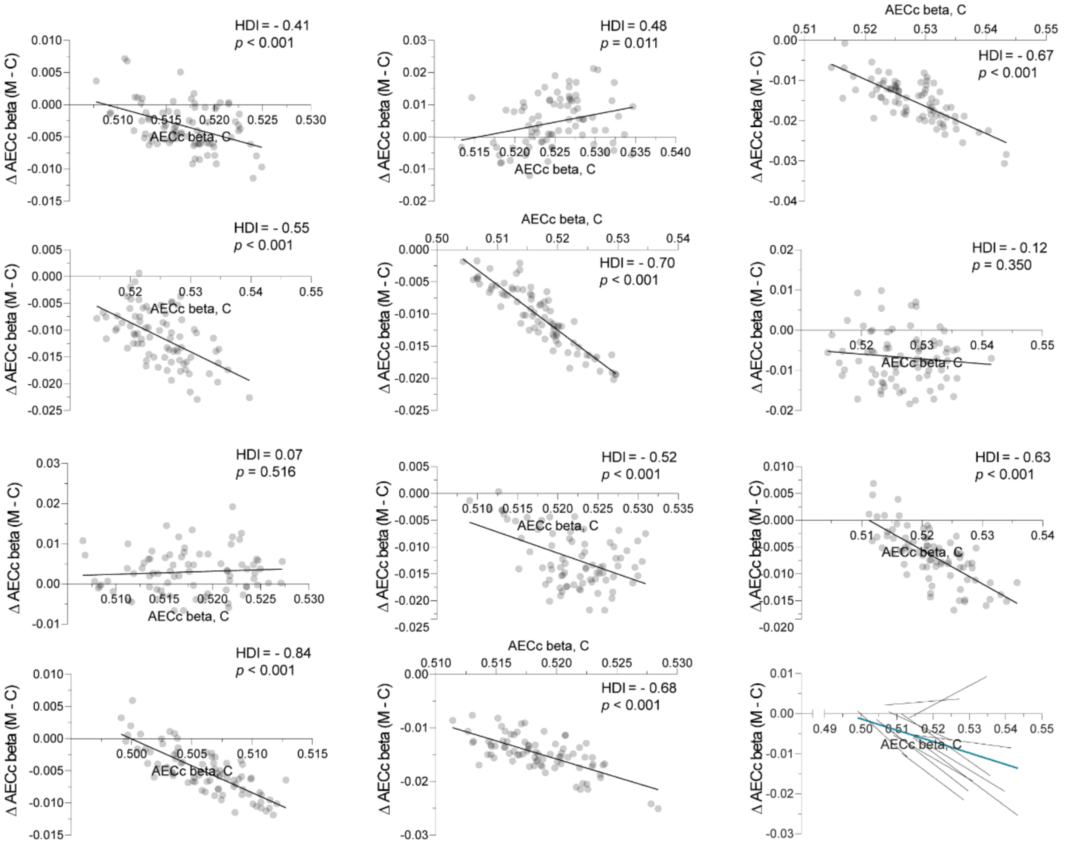

Supplementary Figure 5. Individual beta band AECc hub disruption index for mutation carriers. Each subplot shows the hub disruption index (HDI) results based on beta band AECc for each mutation carrier (n = 11). The difference in regional functional degree (for each of the 80 regions of the AAL atlas) between a mutation carrier (M, n = 1) and (surrogate) control (C, n = 1) is presented on the y-axis as function of the regional functional degree in the surrogate control on the x-axis. The functional degree was computed as the average connectivity strength (measured with AECc in the beta band) between one region and all other brain regions. The (surrogate) control values were obtained by averaging across three healthy controls that matched to one mutation carrier in respect to age and sex. Simple linear regression analyses was conducted to obtain the HDI for each mutation carrier. The bottom right plot shows the 11 linear regression lines for all mutation carriers. The colored line reflects the linear fit when all data points (80 regions for n = 11 mutation carriers and surrogate controls, not shown) are taken into account, indicating the average HDI for mutation carriers. Statistical test details and results are reported in Supplementary Table 6. AECc, amplitude envelope correlation corrected for volume conduction; AAL, automated anatomical labeling.

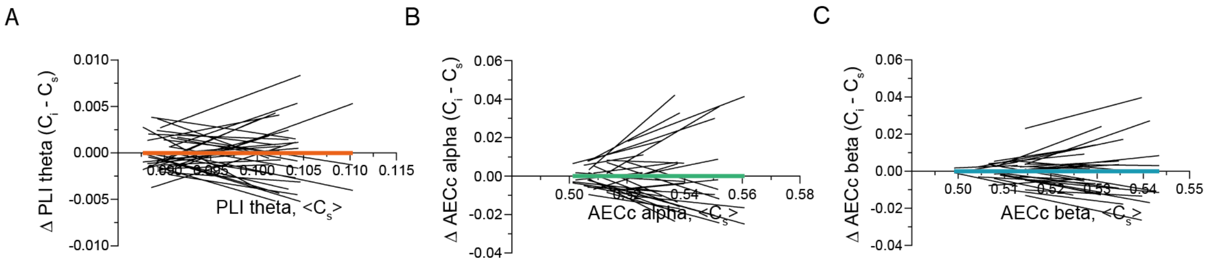

Supplementary Figure 6. **Hub disruption index for controls. A-C)** Each subplot shows the hub disruption index (HDI) results for controls for theta band PLI (A), alpha (B) and beta band AECc (C). The linear regression lines were fit over the differences in regional functional degree between each control (C_i, *n* = 33) and the corresponding (surrogate) control (C_s, total *n* = 11) as a function of the regional degree in the (surrogate) control. The surrogate control values were obtained by taking the average of the three healthy subjects that matched in respect to age and sex to one mutation carrier. Colored lines reflect the linear fit when all data points (80 ROIs for *n* = 33 controls compared to *n* = 11 surrogate controls, not shown) are taken into account, indicating the average HDI for controls. PLI, phase lag index; AECc, amplitude envelope correlation corrected for volume conduction.

References

1. Goldman JS, Hahn SE, Catania JW*, et al*. Genetic counseling and testing for Alzheimer disease: joint practice guidelines of the American College of Medical Genetics and the National Society of Genetic Counselors. *Genet Med*. Jun 2011;13(6):597-605. doi:10.1097/GIM.0b013e31821d69b8
2. Aalten P, Ramakers IH, Biessels GJ*, et al*. The Dutch Parelsnoer Institute--Neurodegenerative diseases; methods, design and baseline results. *BMC Neurol*. Dec 31 2014;14:254. doi:10.1186/s12883-014-0254-4
3. Rattanabannakit C, Risacher SL, Gao S*, et al*. The Cognitive Change Index as a Measure of Self and Informant Perception of Cognitive Decline: Relation to Neuropsychological Tests. *J Alzheimers Dis*. 2016;51(4):1145-55. doi:10.3233/JAD-150729
4. Sikkes SA, Knol DL, Pijnenburg YA, de Lange-de Klerk ES, Uitdehaag BM, Scheltens P. Validation of the Amsterdam IADL Questionnaire(c), a new tool to measure instrumental activities of daily living in dementia. *Neuroepidemiology*. 2013;41(1):35-41. doi:10.1159/000346277
5. Spinhoven P, Ormel J, Sloekers PP, Kempen GI, Speckens AE, Van Hemert AM. A validation study of the Hospital Anxiety and Depression Scale (HADS) in different groups of Dutch subjects. *Psychol Med*. Mar 1997;27(2):363-70. doi:10.1017/s0033291796004382
6. Folstein MF, Folstein SE, McHugh PR. "Mini-mental state". A practical method for grading the cognitive state of patients for the clinician. *J Psychiatr Res*. Nov 1975;12(3):189-98. doi:10.1016/0022-3956(75)90026-6
7. Schmand B. HP, de Koning I. Normen van psychologische tests voor gebruik in de klinische neuropsychologie. Nederlands Instituut van Psychologen. https://www.psynip.nl/secties/sectie-neuropsychologie/
8. Reise SP, Waller NG. Item response theory and clinical measurement. *Annu Rev Clin Psychol*. 2009;5:27-48. doi:10.1146/annurev.clinpsy.032408.153553
9. Sikkes SA, Pijnenburg YA, Knol DL, de Lange-de Klerk ES, Scheltens P, Uitdehaag BM. Assessment of instrumental activities of daily living in dementia: diagnostic value of the Amsterdam Instrumental Activities of Daily Living Questionnaire. *J Geriatr Psychiatry Neurol*. Dec 2013;26(4):244-50. doi:10.1177/0891988713509139
10. van der Flier WM, Pijnenburg YA, Prins N*, et al*. Optimizing patient care and research: the Amsterdam Dementia Cohort. *J Alzheimers Dis*. 2014;41(1):313-27. doi:10.3233/JAD-132306
11. Scheltens P, Leys D, Barkhof F*, et al*. Atrophy of medial temporal lobes on MRI in "probable" Alzheimer's disease and normal ageing: diagnostic value and neuropsychological correlates. *J Neurol Neurosurg Psychiatry*. Oct 1992;55(10):967-72. doi:10.1136/jnnp.55.10.967
12. Koedam EL, Lehmann M, van der Flier WM*, et al*. Visual assessment of posterior atrophy development of a MRI rating scale. *Eur Radiol*. Dec 2011;21(12):2618-25. doi:10.1007/s00330-011-2205-4
13. Lehmann M, Koedam EL, Barnes J*, et al*. Posterior cerebral atrophy in the absence of medial temporal lobe atrophy in pathologically-confirmed Alzheimer's disease. *Neurobiol Aging*. Mar 2012;33(3):627 e1-627 e12. doi:10.1016/j.neurobiolaging.2011.04.003
14. Pasquier F, Leys D, Weerts JG, Mounier-Vehier F, Barkhof F, Scheltens P. Inter- and intraobserver reproducibility of cerebral atrophy assessment on MRI scans with hemispheric infarcts. *Eur Neurol*. 1996;36(5):268-72. doi:10.1159/000117270
15. Fazekas F, Chawluk JB, Alavi A, Hurtig HI, Zimmerman RA. MR signal abnormalities at 1.5 T in Alzheimer's dementia and normal aging. *AJR Am J Roentgenol*. Aug 1987;149(2):351-6. doi:10.2214/ajr.149.2.351
16. Cordonnier C, van der Flier WM, Sluimer JD, Leys D, Barkhof F, Scheltens P. Prevalence and severity of microbleeds in a memory clinic setting. *Neurology*. May 9 2006;66(9):1356-60. doi:10.1212/01.wnl.0000210535.20297.ae
17. Tzourio-Mazoyer N, Landeau B, Papathanassiou D*, et al*. Automated anatomical labeling of activations in SPM using a macroscopic anatomical parcellation of the MNI MRI single-subject brain. *Neuroimage*. Jan 2002;15(1):273-89. doi:10.1006/nimg.2001.0978

18. Gong G, He Y, Concha L*, et al*. Mapping anatomical connectivity patterns of human cerebral cortex using in vivo diffusion tensor imaging tractography. *Cereb Cortex*. Mar 2009;19(3):524-36. doi:10.1093/cercor/bhn102
